# Understanding the Molecular Mechanisms of Incomptine A in Treating Non-Hodgkin Lymphoma Associated with U-937 Cells: Bioinformatics Approaches, Part I

**DOI:** 10.3390/ph18010005

**Published:** 2024-12-24

**Authors:** Fernando Calzada, Normand García-Hernández, Elihú Bautista, José Manuel Sánchez-López, Miguel Valdes, Claudia Velázquez, Elizabeth Barbosa

**Affiliations:** 1 Unidad de Investigación Médica en Farmacología, UMAE Hospital de Especialidades, 2° Piso CORSE, Centro Médico Nacional Siglo XXI, Instituto Mexicano del Seguro Social, Av. Cuauhtémoc 330, Col. Doctores, Mexico City 06725, Mexico; 2 Unidad de Investigación Médica en Genética Humana, UMAE Hospital Pediatría 2º Piso, Centro Médico Nacional Siglo XXI, Instituto Mexicano del Seguro Social, Av. Cuauhtémoc 330, Col. Doctores, Mexico City 06725, Mexico; cienciaflosan@gmail.com; 3 CONAHCYT-División de Biología Molecular, Instituto Potosino de Investigación Científica y Tecnológica A. C, San Luis Potosí 78216, Mexico; francisco.bautista@ipicyt.edu.mx; 4 Instituto Politécnico Nacional, Sección de Estudios de Posgrado e Investigación, Escuela Superior de Medicina, Plan de San Luis y Salvador Díaz Mirón S/N, Col. Casco de Santo Tomás, Miguel Hidalgo, Mexico City 11340, Mexico; valdesguevaramiguel@gmail.com (M.V.); rebc78@yahoo.com.mx (E.B.); 5 Área Académica de Farmacia, Instituto de Ciencias de la Salud, Universidad Autonoma del Estado de Hidalgo, Circuito exHacienda La Concepción S/N, Carretera Pachuca-Atocpan, San Agustin Tlaxiaca 42076, Mexico; cvg09@yahoo.com

**Keywords:** incomptine A, sesquiterpene lactone, *Decachaeta incompta*, cancer, non-Hodgkin lymphoma, TMT, gene ontology, Reactome, KEGG pathway, molecular docking

## Abstract

**Background**: Incomptine A (**IA**) has been reported to have cytotoxic activity in non-Hodgkin lymphoma cancer cell lines and have effects on U-937 cells, including the induction of apoptosis, the production of reactive oxygen species, and the inhibition of glycolytic enzymes. Also, **IA** has cytotoxic activity in the triple-negative subtypes, HER2+, and luminal A of breast cancer cells, with its properties being associated with an effect on the antiapoptotic function of Hexokinase II (HKII). **Objectives**: In this research, we reviewed the altered levels of proteins present in the lymph nodes of male Balb/c mice inoculated with U-937 cells and treated with **IA** or methotrexate, as well as mice only inoculated with cancer cells. **Methods**: Five approaches, including Tandem Mass Tag (TMT), Gene ontology (GO), Reactome, KEGG pathway analysis, and molecular docking, were used. **Results**: TMT showed that 74 proteins were differentially expressed, out of which 12 presented overexpression (FC ≥ 1.5) and 62 were under expressed (FC ≤ 0.67). In general, the TMT approach showed that **IA** had a better effect on proteins than methotrexate. Gene ontology, Reactome, and KEGG pathway analysis showed that proteins with altered levels may be implicated in several processes, including gene silencing by RNA, oxidative phosphorylation, glycolysis/gluconeogenesis, cytoskeleton organization, and ATP metabolic and energetic processes. The molecular docking analysis, which used 23 altered proteins as targets, revealed that **IA** interacted with all the proteins used. **Conclusions**: The results obtained using the five bioinformatic approaches provide information and show that **IA** could be used to treat non-Hodgkin lymphoma induced with the U-937 cell line. Also, it could provide a basis for future research and the development of clinical trials.

## 1. Introduction

**Figure 1:**
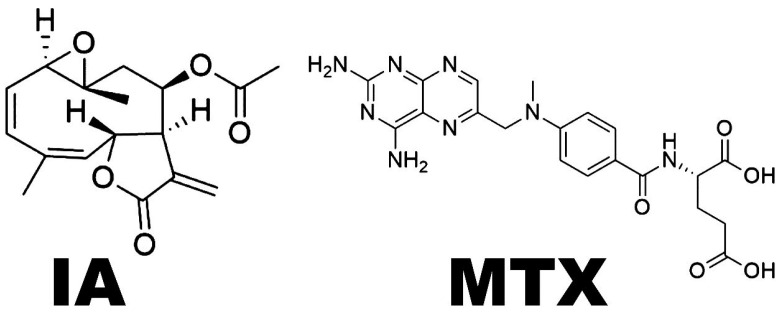
Sesquiterpene lactone, incomptine A (**IA**) and the NHL drug methotrexate (**MTX**).

Cancer is currently one of the main contributors to mortality around the world, and if trends continue, cases will double by 2070 relative to 2020. In this sense, new therapeutic strategies may reduce the prevalence and number of future cases, impacting public health and providing economic benefits [1]. The GLOBOCAN database reported that, in adults, non-Hodgkin lymphomas (NHLs) were responsible for 544,352 new cases and 259,793 deaths worldwide in 2020; among these, 147,217 cases were males and 112,576 were females [2]. In Mexico, until 2021, the epidemiological registry reported that NHLs were the ninth most common cause of cancer for both genders and were the fourth most common cause of death among malignant tumors of lymphatic tissue, blood-forming organs, and related. NHLs include several types of lymphoid tissue neoplasms that develop in the lymph nodes or organs of the human body, induced by the malignant transformation of B or T or NK cells and characterized by an increased proliferation and/or reduction in apoptosis [3,4,5,6]. Treatment usually differs according to the clinical characteristics, but generally includes hematopoietic stem cell transplantation, radiation therapy, systemic anticancer therapy, immune-oncology, and precision medicine. In the case of systemic anticancer therapy (chemotherapy), the anticancer drugs currently used show several strong side effects; for example, in Mexico, methotrexate (**MTX**) (Figure 1) is considered the most important for treating NHLs. It causes genotoxic damage and side effects such as kidney injury, nephrotoxicity, myelosuppression, hepatotoxicity, dermatologic toxicity, and mucositis [3,7]. Therefore, it is important to understand the biology of NHLs and search for new treatments that are safer, more effective, and specific against NHLs. In this context, the incorporation of proteomic studies with bioinformatic strategies may lead to improvements in the obtention of novel, effective and safer anticancer drugs, specifically for the treatment of NHLs [8,9].

Incomptine A (**IA**) (Figure 1), a secondary metabolite obtained from *Decachaeta incompta* (DC) King and Robinson, has been reported to have a broad range of pharmacological activities, including phytotoxic, anti-propulsive, antiprotozoal, spermatic, and antibacterial effects. In addition, it exhibits cytotoxic effects on HL-60, K-562, REH, Farage, SU-DHL-2, REC-1, HER2+ and U-937 cell lines. In the case of U-937 cells, reactive oxygen species (ROS) production and the induction of apoptosis lead to redox imbalance. Also, it has been reported in in vitro studies on U-937 cells using TMT (isobaric tags for relative quantitation) and molecular docking approaches that the antitumor potential of **IA** may be due to the inhibition of cytoskeleton proteins and glycolytic enzymes such as LDHA, LDHB and ALDOA [5,10]. In this context, other sesquiterpene lactones (SLs) have been reported to induce the downregulation of Bcl-2 anti-apoptotic proteins, reduce glutathione depletion, change redox cell balance and impact NF-kB, STAT3 or signaling pathways; these activities affect protein expression during the cell cycle, proliferation, metastasis or cellular invasion, the production of free radicals, and induce apoptosis [10,11,12]. The cytotoxic activity of SLs including **IA** has been associated with the 3-methylenedihydrofuran-2(3*H*)-one moiety, which confers its power as a Michael acceptor and interacts with sulfhydryl groups of proteins [11]. Herein, we examined the antitumor activity of **IA** against NHLs by measuring its effects on the proteins in the lymph nodes of Balb/c mice inoculated with U-937 cells, compared to positive controls administered methotrexate. To investigate the complexity of proteomics from lymph nodes mass in the context of differential protein expression, these were analyzed using five approaches; first, TMT coupled to UPLC-MS/MS (ultra-high performance liquid chromatography–tandem mass spectrometry) was performed [13,14]. Then, all altered proteins were subjected to Gene ontology, Reactome, and KEGG pathway analyses. In addition, **IA** was submitted for molecular docking analysis using the 23 target DEPs (differentially expressed proteins).

## 2. Results

### 2.1. Isolation of Incomptine A (**IA**) of the Leaves from Decachaeta Incompta

**Figure 2:**
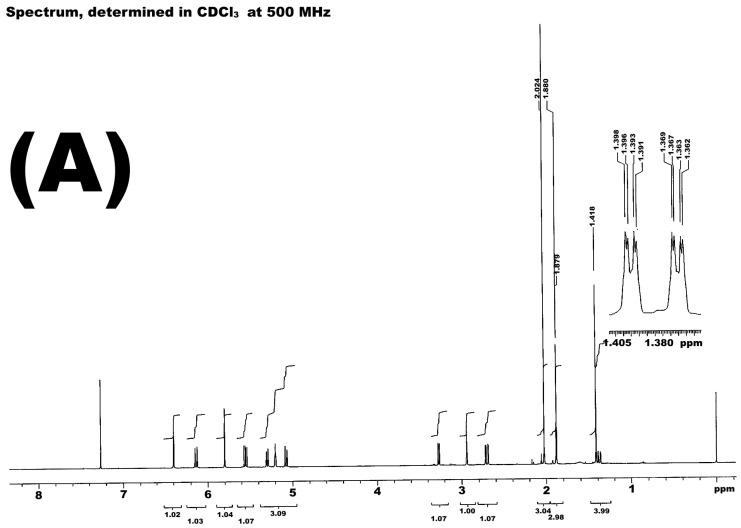

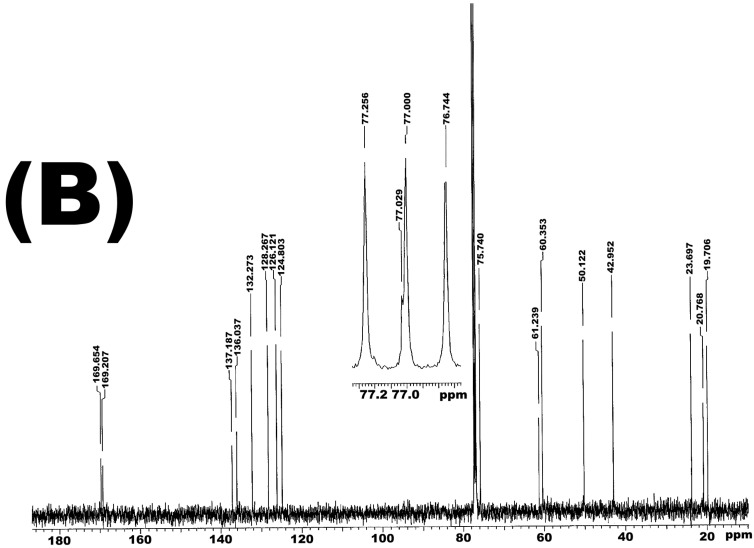
NMR spectra from incomptine A (**IA**), ^1^H-NMR (**A**) and ^13^C-NMR (**B**).

**Figure 3:**
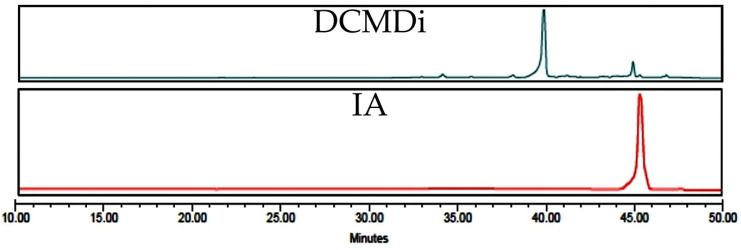
HPLC coupled to diode-array detection (240 nm) of dichloromethane extract from *Decachaeta incompta* (**DCMDi**) and incomptine A (**IA**).

The leaves of *Decachaeta incompta*, collected at Portillo de Nejapa, State of Oaxaca, Mexico, were extracted with dichloromethane to obtain 22.9 g of a brown and pasty extract (6.5%). The organic extract from the *D. incompta* leaves was purified via column chromatography to yield (370.4 mg) of a germacrane-type sesquiterpene lactone known as incomptine A (**IA**). It was identified according to its ^13^C and ^1^H NMR spectra (Figure 2) and a comparison of the retention time in HPLC-DAD (Figure 3), with the authentic sample provided by Dr. E. Bautista [15].

### 2.2. Anti-Lymphoma Activity of Incomptine A (**IA**)

**Figure 4:**
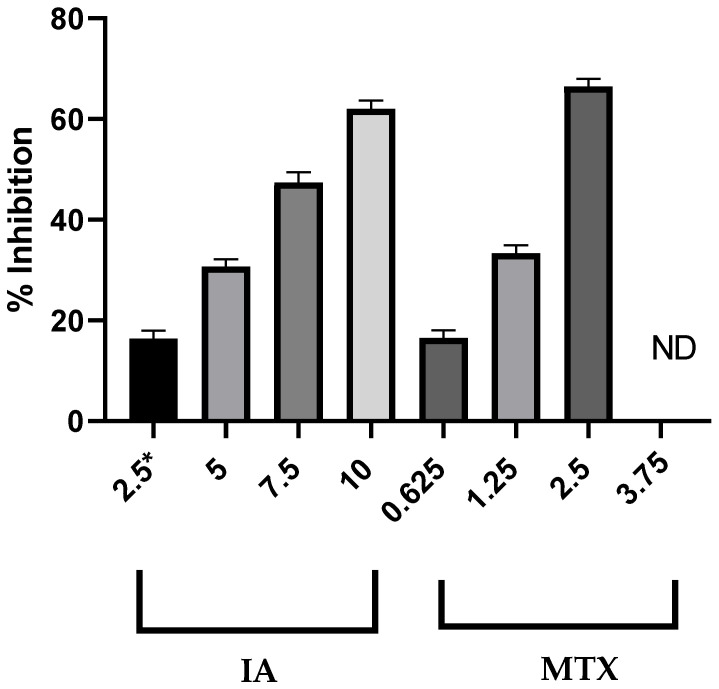
Anti-lymphoma activity of incomptine A (**IA**) and methotrexate (**MTX**). ND: Not determined, **MTX** at dose of 3.75 mg/kg resulted in 100% mortality in male Balb/c mice. * Doses (mg/kg).

Table 1 shows the anti-lymphoma activity in male Balb/c mice administered DCMDi, **IA**, and **MTX;** these had important antitumor effects, with EC_50_ values of 80 mg/kg, 16.42 μmol/kg, and 2.75 μmol/kg, respectively. The activity of **IA** was dose-dependent (Figure 4). The effect of **IA** was similar to **MTX**. It is important to point out that **MTX** at doses of 3.75 mg/kg causes 100% mortality in mice.

### 2.3. Identification of Non-Hodgkin Lymphoma Differentially Expressed Proteins Induced by Incomptine A (**IA**)

To understand the molecular mechanisms of incomptine A (**IA**) in non-Hodgkin lymphoma, a proteomic approach was used. The differentially expressed proteins (DEPs) in the lymph node samples obtained from male Balb/c mice inoculated with U-937 cells and treated with **IA** or **MTX,** as well as the lymph nodes of mice only inoculated with cancer cells, were characterized by TMT coupled to UPLC-MS/MS [16,17,18,19,20]. Six raw MS files with 20,453 peptides were analyzed and searched against the mouse protein database. In total, 2717 proteins were identified in the samples. Proteins with altered levels in the lymph nodes were quantified based on the intensity of the TMT labels for peptides. The screening criteria for the relative quantitation of proteins (Table 2) were fold-change > 1.5 (upregulated) or <0.67 (downregulated).

In total, 74 significantly differentially expressed proteins were obtained, including **IA** and **MTX**-treated samples. Among these, 14 were only affected by **IA** (Table 3) and 60 overlapped in **IA** and **MTX** (Table 4). Table 3 shows that **IA** induced downregulation in five proteins and upregulation in nine. In contrast, these did not change in the proteins treated with **MTX**. In addition, of the 60 proteins affected by **IA** and **MTX** (Table 4), three proteins were upregulated and the remaining proteins were downregulated.

### 2.4. Bioinformatics Analysis

#### 2.4.1. Results of the Enrichment Analysis by Reactome, and g: Profiler

**Figure 5:**
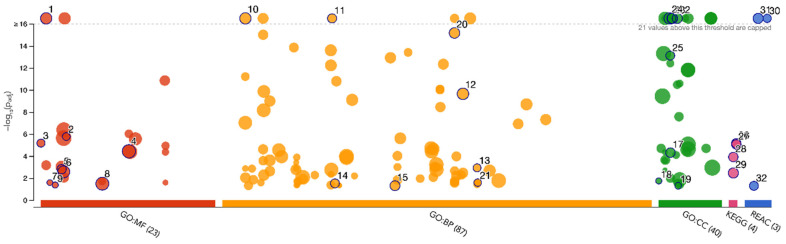
GO, KEGG and REAC enriched terms. GO: MF, gene ontology molecular function; GO: BP, gene ontology biological process; GO:CC, gene ontology cellular component.

**Figure 6:**
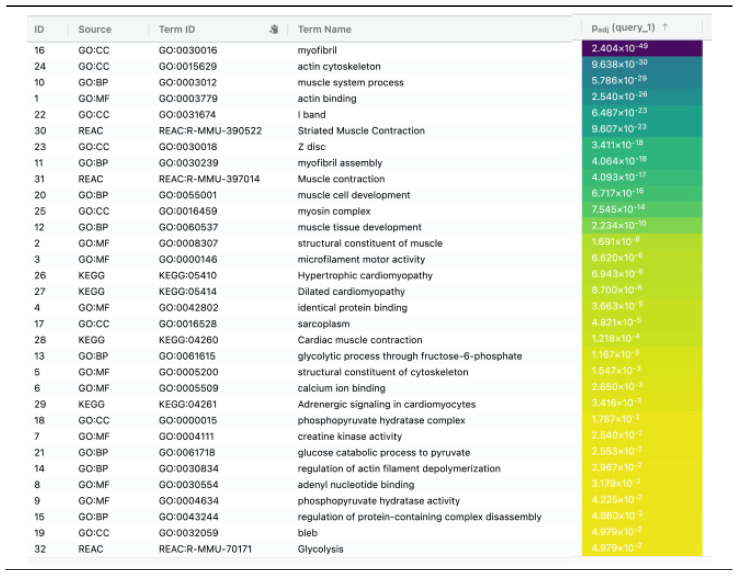
Top 32 significantly changed terms enriched by GO, KEGG, and Reactome databases.

To visually observe the enrichment information of the 74 DEPs obtained for the lymph nodes of Balb/c mice inoculated with U-937 cells and treated with **IA**, g: Profiler and Reactome (REAC) analysis was performed. In total, 157 terms were enriched, with 150, 4, and 3 associated with Gene Ontology (GO), Kyoto Encyclopedia of Genes and Genomes (KEGG), and REAC, respectively (Figure 5) [21,22]. In the case of GO, 23, 87, and 40 terms were associated with molecular function (MF), biological process (BP), and cellular component (CC), respectively. The top 32 significantly changed terms enriched by the GO, KEGG, and REAC databases are shown in Figure 6.

**Figure 7:**
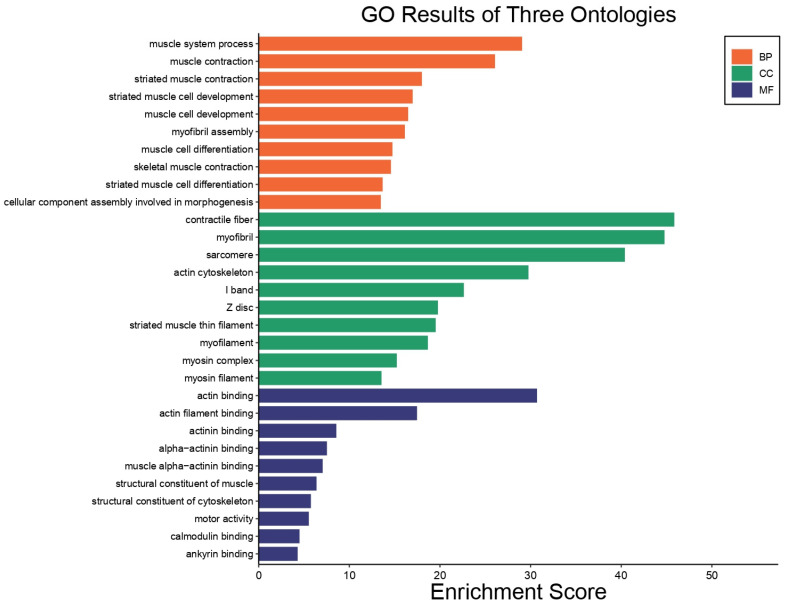
GO enrichment analysis of all DEPs. The top 10 enriched GO terms are listed in the diagram for BP, CC, and MF terms.

In BP, the significantly overrepresented GO, muscle system process, muscle contraction, striated muscle contraction and the cellular component assembly involved in morphogenesis were among the top 10 processes (Figure 7). In CC terms, contractile fiber, myofibril, sarcomere and the actin cytoskeleton were among the top 10 cellular components. Finally, actin binding, actin filament binding, actinin binding and the structural constituents of cytoskeleton and motor activity were among the top 10 MF terms.

The list of the representative processes arranged by significance value, labeled by ID (number in the general list of the process), resource (database with which the process was enriched), term ID (identification of the process according to each platform), term name (name of the enriched process according to each database) and adj-*p*-value (significance value of the process) shown with the number and color scale and arranged in a descending order.

#### 2.4.2. Results of Protein–Protein Interactions Network Analysis

**Figure 8:**
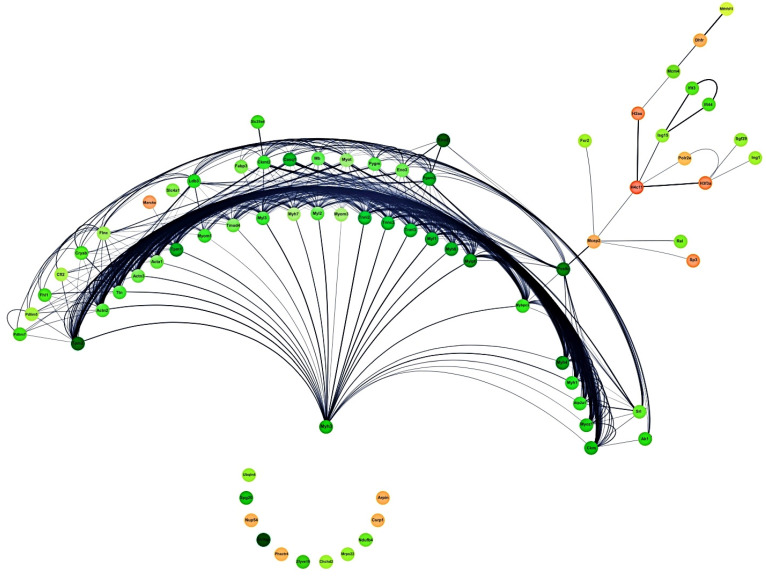
The 74 differentially expressed proteins involved in NHL. The orange color shows upregulated genes and the green color shows downregulated genes.

In order to identify the most important clusters affected by **IA** and their core targets in the treatment of non-Hodgkin lymphoma (NHL) induced on Balb/c mice inoculated with U-937 cells, 74 DEPs, including 12 upregulated proteins and 62 downregulated proteins listed in Table 3 and Table 4, were analyzed by the protein–protein interaction network (Figure 8). Among, the three most important targets of **IA** in the treatment of NHL in mice were Myh3, Eno2, and H4c11 (Histone H4 or Hist1h4a). Myh3 and Eno2 were two downregulated proteins and H4c11 was an upregulated protein. Among these, Myh3 was the most important protein, directly or indirectly interacting with another 62 proteins. H4c11 and Eno2 showed 15 and 5 interactions with other proteins. In addition, 11 DEPs, including four upregulated proteins and seven downregulated proteins, did not show any interaction with other proteins.

#### 2.4.3. Results of the GO and KEEG Enrichment Analysis

**Figure 9:**
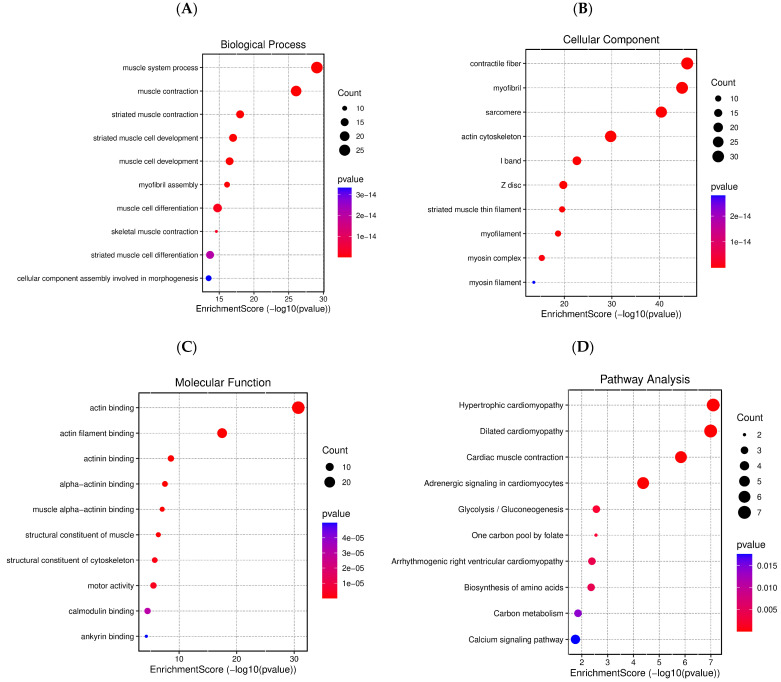
Enriched pathways in non-Hodgkin lymphoma in mice. (**A**) Gene ontology in terms of biological processes, (**B**) gene ontology in terms of cellular components, (**C**) gene ontology in terms of molecular function, (**D**): KEGG.

To understand the biological changes during NHL in mice, analyses using the KEGG and DAVID databases were performed. Figure 9 shows the top 10 GO:BP, GO:CC, GO:MF, and KEGG terms affected by **IA** in NHL. GO:BP includes muscle system processes and development (A). The GO:CC annotation was related to contractile fiber, myofibril, sarcomere, actin cytoskeleton, and I band (B). The MF was associated with actin (C). KEGG analysis was related with glycolysis/gluconeogenesis, one carbon pool by folate, right ventricular arrhythmogenic cardiomyopathy, the biosynthesis of amino acids, and carbon metabolism, as well as the calcium signaling pathway. These pathways represented the 10 main biochemical pathways and signal transduction pathways that are affected by DEPs.

**Figure 10:**
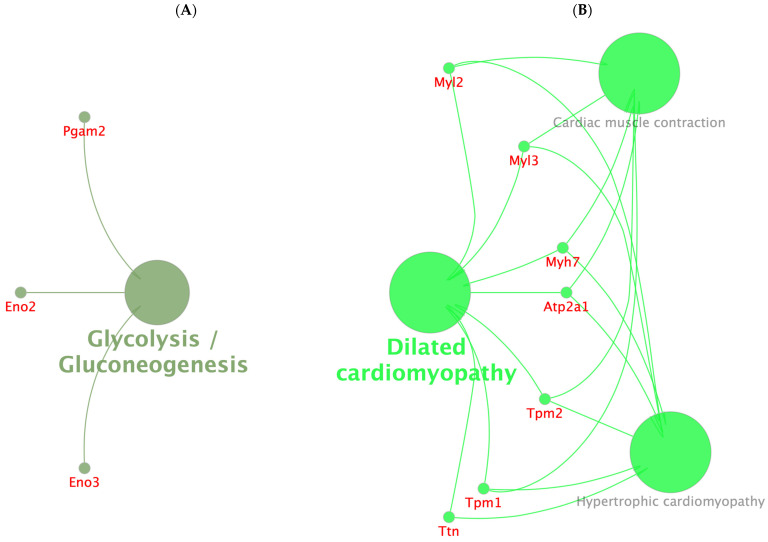
Correlation between DEPs and four of the top pathways in KEGG analysis: (**A**) glycolysis/gluconeogenesis; (**B**) dilated cardiomyopathy, cardiac muscle contraction, and hypertrophic cardiomyopathy.

Among these glycolysis/gluconeogenesis pathways (Figure 10), three downregulated proteins, including Eno2, Eno3, and Pgam2, were enriched (A). In the cases of dilated cardiomyopathy, cardiac muscle contraction, and hypertrophic cardiomyopathy (B), seven downregulated proteins were enriched, including Myl2, Myl3, Myh7, Atp2a1, Tpm1, Tpm2, and Ttn (Figure 10).

#### 2.4.4. Results of the REACTOME Enrichment Analysis

**Figure 11:**
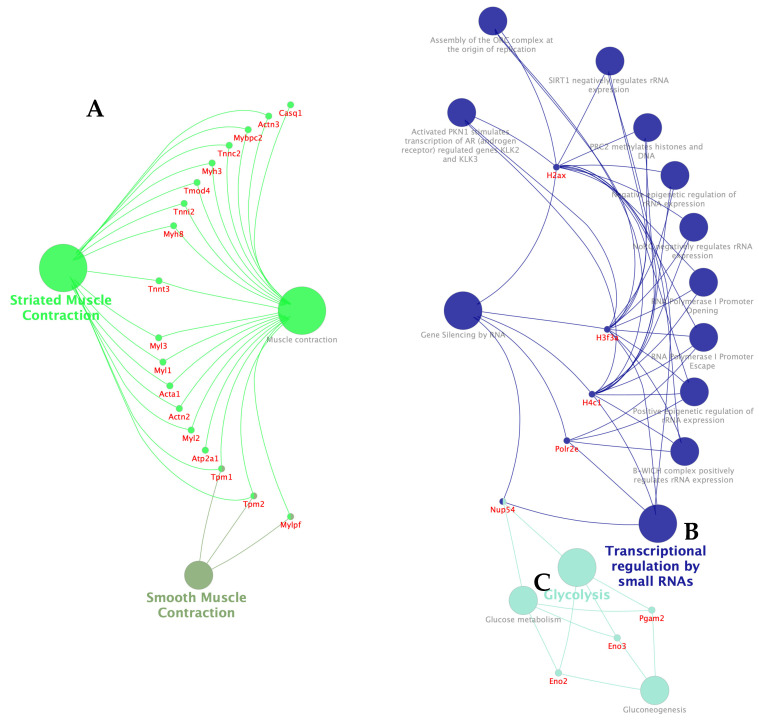
Correlation between DEPs and top four pathways in Reactome analysis: (**A**) Striated muscle contraction and muscle contraction; (**B**) transcriptional regulation by small RNAs and (**C**) glycolysis/glucose metabolism/gluconeogenesis.

The analysis of REAC pathways showed that the glycolysis, striated muscle contraction, muscle contraction, and transcriptional regulation by small RNAs (Figure 11) pathways were enriched in several DEPs. Among these, striated muscle contraction and muscle contraction were enriched in 18 downregulated genes, including Casq1, Actn2, Actn3, Mybpc2, Tnnc2, Tnnt3, Myh3, Myh8, Myl1, Acta1, Tmod4, Tnni2, Mylpf, Myl2, Myl3, Myh7, Atp2a1, Tpm1, and Tpm2 (A). Transcriptional regulation by small RNAs was enriched in five upregulated proteins, including Nup54, Polr2e, H4c11 (Hist1h4a or Histone H4), H3f3a, and H2ax (H2afx) (B). In the case of glycolysis, three downregulated proteins were enriched, including Eno2, Eno3, and Pgam2 (C).

#### 2.4.5. Molecular Docking Studies of Incomptine A (**IA**) on 21 Proteins with Altered Levels in LNH in Mice

**Figure 12:**
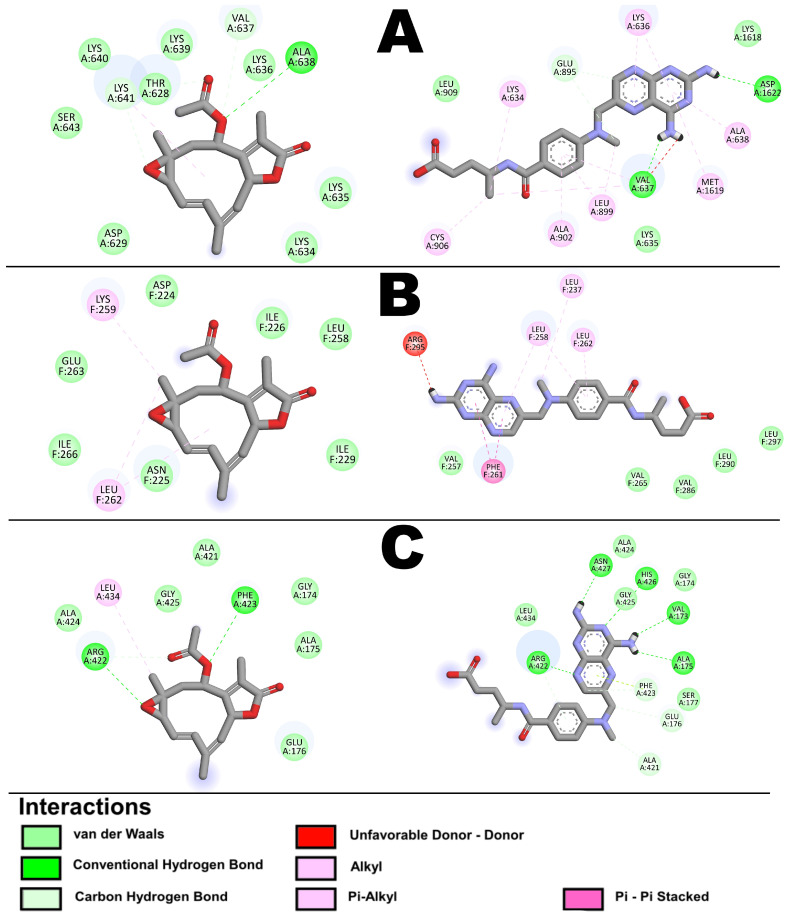
Results of molecular docking on proteins, 2D representation of interactions between Incomptine A (**IA**) and Methotrexate (**MTX**), (**A**) Myosin-3 (Myh-3), (**B**) Histone H4 (Hist1h4a or H4c11), and (**C**) Gamma enolase (Eno2).

Considering the previous results shown here, including those of the TMT, GO, KEEG, and REAC analyses, a molecular docking study was carried out to provide additional information about the interaction between incomptine A (**IA**) or methotrexate (**MTX**) with 23 DEPs (Supplementary Material) [23,24]. Molecular docking showed that **IA** has a better affinity for the proteins Myosin-3 (Myh3, Figure 12A), Myozenin-1 (Myoz1), Histone H2-X (H2afx), Histone H3.3 (H3f3a), Histone H4 (Hist1h4a or H4c11, Figure 12B), Methyl-CPG-binding protein 2 (Mecp2), Myosins (My13, My12, My1pf, and Myh8), Myosin-binding protein C (Mybpc2), and Monofunctional C1-tetrahydrofolate dehydrogenase fast-type (Marcks), with ∆G values of −5.35, −4.7, −4.25, −4.47,−4.71, −5.33, −5.22, −5.1, −5.72, −6.09, −4.26, and −6.95 kcal/mol, respectively; these bind to several amino acid residues, principally with polar interactions (Table 5, Supplementary Material). In relation to ADP/ATP translocase (S1c25a4), Alpha-actin 3 (Actn3), 28S ribosomal protein S22 (Mrps22), Actin alpha skeletal muscle (Acta1), Alpha-actinin-2 (Actn2), Beta-enolase (Eno3), Gamma enolase (Eno2, Figure 12C), Myosin-1 (Myh1), Myosin-4 (Myh4), Myosin-7 (Myh7), and Dihydrofolate reductase (Dhfr), methotrexate shows a better affinity, with ∆G values of −5.24, −4.87, −4.95, −5.73, −6.12, −7.31, −6.47, −5.23, −5.65, −4.68, and −8.92 kcal/mol, respectively (Supplementary Material).

## 3. Discussion

Lymphomas are the most common blood cancer, and principally affect lymphocytes. In this sense, there are three main classes of lymphomas, namely small lymphocytic lymphoma, Hodgkin’s lymphoma and NHL. NHL represents the most common hematological malignancy, with nearly 90 types; these are diagnosed in nearly 544,000 people annually, resulting in nearly 259,793 deaths worldwide [25,26].

As part of our continuing research about the potential use of anti-cancer agents obtained from medicinal plants, we aimed to evaluate the anti-lymphoma activity of incomptine A (**IA**), as well to investigate the molecular effects of **IA** in a model of non-Hodgkin lymphoma in male Balb/c mice. We first evaluated the anti-lymphoma activity of IA in male Balb/c mice inoculated with U-937 cells and treated with **IA** or methotrexate (**MTX**). Then, we analyzed the lymph nodes obtained from male Balb/c mice using five approaches. First, TMT coupled with UPLC-MS/MS was employed [13,14]. Afterwards, all altered proteins were subjected to Gene ontology, Reactome, KEGG pathway, and molecular docking analyses [16,17,18,19,20,21,22]. Finally, **IA** was submitted for molecular docking analysis using 23 DEPs as targets [23,24].

In relation to anti-lymphoma activity of DCMDi, sequential chromatography in a column over silica gel was first performed to afford a sesquiterpene lactone that was identified as **IA** [15]. Then, **IA**, **MTX**, and DCMDi were evaluated in an anti-lymphoma assay. The activity of **IA**, **MTX**, and DCMDi showed that the anti-lymphoma activity of DCMDi (EC_50_ 80.0 mg/kg) was similar and consistent with data recently reported (EC_50_ 75.0 mg/kg) for this extract in a model of non-Hodgkin lymphoma in female Balb/c mice. In addition, this was in agreement with other extracts obtained from medicinal plants with important anti-lymphoma activity, such as *Annona diversifolia* and *Schinus molle* [6]. **IA** showed dose-dependent anti-lymphoma activity, being more active than DCMDi and similar to **MTX**. In addition, these data were similar and in agreement with the recently reported data for **IA** in a model of non-Hodgkin lymphoma in female Balb/c mice [6]. The results showed that the anti-lymphoma activity of DCMDi and **IA** is not dependent on sex. Also, the activity of **IA** was comparable to other secondary metabolites with significant anti-lymphoma activity, including rutin, geranylgeraniol, phytol and farnesyl acetate [27]. Also, the anti-lymphoma activity of **IA** indicated that SLs could be used as secondary metabolites, with the potential to be developed into new anticancer drugs [10]. In this context, the SLs are compounds that exhibited several biological properties, including phytotoxic, antidiabetic, antimicrobial, and antitumor effects [15,28,29,30]. In this sense, **IA** belongs to a group of SLs that have been reported to have significant anticancer activity associated with the presence of a *α*-methylene-γ-lactone moiety. These SLs include eupatolide, deoxyelephantopin, dehydrocostus lactone, and parthenolide. In the case of parthenolide, it is currently being assayed in cancer clinical trials [10,31,32,33]. It is important to mention that **MTX** caused 100% mortality in male Balb/c mice at doses from 3.75 mg/kg; this result is in agreement with the toxicity previously reported in humans and mice [7,11].

The next step in our investigation was understanding the molecular effects of **IA** on NHL in a mice model induced with U-937 cells. Lymph nodes were removed after male Balb/c mice had previously been inoculated with U-937 cells for 30 days and treated with IA (5 mg/kg) or methotrexate (1.25 mg/kg) for eight days, as well as mice only inoculated with U-937 cells. In this sense, TMT coupled with LC-MS/MS was performed to identify 2717 reliable proteins from lymph nodes, with 74 of them being differentially expressed. Among these, 14 proteins were only altered by **IA** and 60 proteins were altered by **IA** and **MTX**. In this sense, **IA** downregulated a total of 62 proteins and upregulated 12 proteins. **MTX** downregulated a total of 57 proteins and upregulated 3 proteins. In general, **IA** induced more downregulation and upregulation than **MTX**.

All DEPs affected by the **IA** or **MTX** treatments were subjected to protein–protein interaction analysis, showing that Myosin-3 (Myh3), Gamma enolase (Eno2), and Histone H4 (Hist1h4a or H4c11) were the three most important core proteins (Figure 8). In agreement with the GO, KEGG, and REACTOME analyses, the DEPs may be associated with several biological processes including striated muscle contraction, smooth muscle contraction, muscle contraction, dilated cardiomyopathy, cardiac muscle contraction, hypertrophic cardiomyopathy, transcriptional regulation by small RNAs, gene silencing by RNA, glycolysis, gluconeogenesis, and glucose metabolism (Figure 9, Figure 10 and Figure 11).

In order of importance for its interactions, Myosin-3 (Myh3) showed a direct interaction relationship (Figure 8) with 24 DEPs, including tropomyosins, actinins, myomesins, myosins and troponins, among others [34,35,36,37,38,39]. In addition, it had an indirect interaction relationship with another 38 DEPs (Figure 8). Myh3 can convert chemical energy into mechanical energy through the hydrolysis of ATP. It is present in eukaryotic cells, has a relationship with tumor occurrence and development, and is reportedly upregulated in several cancers, including breast cancer, colon adenocarcinoma, tongue cancer, lung cancer, and prostate cancer [40,41,42,43,44]. Hist1h4a (histone H4 or H4c11) showed a direct interaction relationship with five DEPs, including Mecp2, H2ax, Isg15, Polr2e, and H3f3a (Figure 8). Hist1h4a, a member of the histone family proteins, is a nuclear DNA-binding protein involved in the structure of chromatin, nucleosomes, and the dynamics of all eukaryotic cells. Also, it affects the regulation of gene expression and provides structural support to chromosomes [45]. This histone can be involved in apoptosis and inflammatory tissue responses through the regulation of downstream proteins. The upregulation of Hist1h4a is associated with lung, breast and acute lymphoblastic leukemia cancer [46,47,48]. Eno2 (gamma enolase or neuron specific enolase) showed a direct interaction relationship with five DEPs, including Pgam2, Eno3, Pavalb, Ak1, and Pygm (Figure 8). Eno2 is a general tumor marker for neuroendocrine tumors, and is used to aid in cancer diagnosis, prognosis, and characterization. Its enzyme catalyzes the conversion of 2-phosphoglycerate into phosphoenolpyruvate [49]. As an oncogene, Eno2 is associated with increased cell growth, metastasis, tumor progression, glucocorticoid resistance, the activation and enrichment of glycolysis, distant metastasis, and increased migration and invasion. A high expression of Eno2 has been reported in breast cancer, acute lymphoblastic leukemia, small cell lung cancer, neuroblastoma, melanoma, retinoblastoma, seminoma, dysgerminoma, malignant phaeochromocytoma, pancreatic ductal adenocarcinoma, prostate cancer, liver cancer, cervical cancer, esophageal cancer, kidney cancer, sarcoma, colorectal cancer, and head and neck squamous cell carcinoma [49,50,51,52,53,54,55,56]. Regarding the remaining DEPs, Myh4, Acta1, Tnnt3, Mb, Ttn, Actn2, Myh7, Myl1, Mybpc2, Myl3, and Tnnc2 are associated with several cancers [41,42,44,46,56].

In our research, 62 DEPs were downregulated in the lymph nodes of animals with NHL and treated with **IA,** including Myh3 and Eno2. It has been reported that the downregulation of Myh3 and Eno2 in cancer patients is a good prognostic tool [40,41,42,43,44,49,50,51,52,53,54,55,56]. Also, when Eno2 is upregulated in several cancers without any treatment, it can be used as a biomarker of illness. In this context, Eno2 was found in this work, suggesting that its protein may be used as a biomarker of NHL [49,50,51,52,53,54,55,56].

In agreement with the GO, Reactome, KEGG pathway, and molecular docking analyses, incomptine A interacted with the proteins Myh3, Eno2, and H4c11. The interactions shown in the molecular docking analysis between **IA** and the DEPs Myh3, Eno2, and H4c11 additionally support the effects of **IA** in several processes, including gene silencing by RNA, oxidative phosphorylation, glycolysis/gluconeogenesis, cytoskeleton organization, and ATP metabolic and energetic processes. In addition, the results indicate that the five approaches used in this work can be employed to reliably find novel targets, processes and mechanisms involved in NHL. Also, they may be used in other research to determine potential targets, find novel biomarkers and examine the processes involved in other cancers.

Finally, assessments such as Western blotting, immunohistochemistry and miRNA expression using qRT-PCR are necessary to validate the results obtained here.

## 4. Materials and Methods

### 4.1. The Plant Material and Isolation of Incomptine A (**IA**)

Compound **IA** was obtained from *Decachaeta incompta* leaves (Asteraceae). The plant material was obtained in the State of Oaxaca, Mexico, in February 2022 and authenticated by a taxonomist at the Herbarium, IMSSM from Instituto Mexicano del Seguro Social, with code number 15311. The phytochemical procedure was carried out according to a protocol previously described [15]. The identification of **IA** was carried out via a comparison with a standard of 99% of, HPLC-DAD, ^1^H-NMR and ^13^C-NMR spectra.

### 4.2. Reagents and Instrumentation

Triethylammonium bicarbonate buffer (1.0 M, pH 8.5 ± 0.1), tris (2-carboxyethyl) phosphine hydrochloride solution (0.5 M, pH 7.0), iodoacetamide (IAA), formic acid (FA), acetonitrile (ACN), methanol (Sigma-Aldrich, San Luis, AZ, USA), trypsin from bovine pancreas (Promega, Madison, WI, USA), ultrapure water (Millipore, Burlington, MA, USA), TMT 6-plex Isobaric Label Reagent were obtained, and a Pierce Quantitative Colorimetric Peptide Assay was performed (Thermo Fisher Science, Waltham, MA, USA). The ultimate 3000 nano UHPLC system coupled online to a Q Exactive HF mass spectrometer equipped with a Nano spray Flex Ion Source (Thermo Scientific, Waltham, MA, USA) was used. The TMT-based Quantification Analytical Service was provided by Creative Proteomics (Shirley, NY, USA).

### 4.3. Cell Culture Conditions

U-937 cells were acquired from the American Type Culture Collection (CRL-1593,2). To develop the mice model, U-937 cells were cultured at 37 °C in RPMI 1640 culture medium (GIBCO Cat: 11875-093) supplemented with 5% fetal bovine serum (GIBCO Cat: 16000044), penicillin (100 U/mL)/streptomycin (100 μg/mL), and 5% CO_2_. In vivo assays were performed using cell cultures at a density of 2.5 × 10^6^ cells in T75 flasks (InvitrogenWaltham, MA, USA).

### 4.4. Animals

To induce the in vivo lymphoma model, we used healthy male Balb/c mice (25 ± 3 g), provided by the animal house of the Centro Médico Nacional Siglo XXI, Instituto Mexicano del Seguro Social. The research received ethical approval from the National Committee of Scientific Research from Instituto Mexicano del Seguro Socia (Approval R-2018-785-111). The mice were maintained in polyvinyl cages at 22 °C under light–dark periods of 12 h, with ad libitum access to food and water, in agreement with Mexican Official Norma, NOM-062-ZOO-1999 [57].

#### Anti-Lymphoma Test

The anti-lymphoma activity test was performed using a method previously described by Calzada et al. [5]. Briefly, the Balb/c mice were divided into eleven groups with 5 mice per group: GNHL1, GNHL2, GNHL3 [GNHL3a, GNHL3b, and GNHL3c], GNHL4 [GNHL4a, GNHL4b, and GNHL4c], and GNHL5 [GNHL5a, GNHL5b, and GNHL5c]). For comparison, GNHL1, designated as the healthy group, was treated with tween 80 in water (2% *v*/*v*); and GNHL2 was used as the negative control, only being inoculated with 1 × 10^6^ U-937 cells. The GNHL3, GNHL4, and GNHL5 groups were injected intraperitoneally with 1 × 10^6^ U-937 cells and treated orally for 9 days with **DEDi** (25, 50, and 100 mg-kg^−1^), incomptine A (**IA**, 2.5, 5.0, 7.5. and 10 mg-kg^−1^), and methotrexate (**MTX**, 1.25, 2.5, and 3.75 mg-kg^−1^), respectively. The animals were maintained under observation for 30 days, with their daily survival and weight recorded. The mice were then sacrificed, and their axillary and inguinal lymph nodes were removed and weighed. The anti-lymphoma activity was determined by comparing the total lymph node weight of each group against the negative control; then, the percentage of inhibition and the 50% effective inhibitory concentration (EC_50_) were calculated. The lymph nodes obtained from the GNHL2, GNHL4, and GNHL5 groups were used in the TMT test.

### 4.5. Non-Hodgkin Lymphoma Protein Expression Induced Through **IA**

To evaluate the changes in the proteome of the non-Hodgkin lymphoma experimental model, we clustered the lymph nodes as pools of all mice in each group, concerning the 5 mg/kg and 1.25 mg/kg **IA** and MTX treatments, respectively.

#### Sample Preparation for TMT-Based Proteomic Analysis

Following each lymph node pool, the next steps were as follows: *Protein obtention:* The tissue was lysed using a TissueLyser after being centrifuged at a low temperature for 15 min at 12,000 rpm, and the protein concentration of the supernatant was determined using a BCA kit. Protein Digestion: First, 100 μg of protein was treated with TCEP (10 mM) at 56 °C for 1 h and then alkylated with IAA (20 mM) at room temperature in the dark for 1 h. After this, free trypsin was added at a ratio of 1:50, and incubated overnight at 37 °C. The peptides were lyophilized to near dryness and re-dissolved with TEAB (100 mM). Peptide Labeling: Anhydrous acetonitrile (41 μL) was added to each peptide sample, shaken for 5 min and centrifuged. The supernatant was transferred to the TMT Reagent vial using the following: (C-)/TMT, **MTX**/TMT, and **IA**/TMT. The reaction mixture was incubated at room temperature for 1 h; then, 5% hydroxylamine (8 μL) was added and incubated for another 15 min. Each sample was subjected to HPLC.

### 4.6. Nano MS Analyses

The TMT labeled peptides were analyzed by a LC-MS/MS Ultimate 3000 nano UHPLC system coupled online to a Q Exactive HF mass spectrometer equipped with a Nano spray Flex Ion Source (Thermo Scientific, Waltham, MA, USA). Briefly, 2 μg of sample was injected onto a trap column (PepMap C18, 100 Å, 100 μm × 2 cm, 5 μm) after being subjected to fractionation on an analytical column (PepMap C18, 100 Å, 75 μm × 50 cm, 2 μm). A linear gradient was used: 5 to 7% buffer B in 2 min, from 7% to 20% buffer B in 80 min, from 20% to 40% buffer B in 35 min, then from 40% to 90% buffer B in 4 min. Mobile phase: A: 0.1% formic acid in water; B: 0.1% formic acid in 80% acetonitrile. For TMT-labeled samples, a full scan was carried out between 350 and 1650 *m*/*z*, at a resolution of 200, at 120,000 Th, and with a gain control of 3 to 6. The MS/MS scan was operated in Top 15 mode using the following settings: resolution 200 at 30,000 Th; automatic gain control target 1e5; charge sate exclusion: unassigned, 1, >6; dynamic exclusion 40 s, normalized collision energy at 32%; and isolation window of 1.2 Th.

### 4.7. Protein Identification

The raw MS files of 6 replicates were analyzed and compared against the mouse protein database using Maxquant (1.6.2.6). The parameters were set as follows: The protein modifications were carbamidomethylation (C, fixed), oxidation (M, variable) and TMT-6Plex. Enzyme specificity was set to trypsin, the maximum missed cleavages were set to 2, the precursor ion mass tolerance was set to 10 ppm, and the MS/MS tolerance was 0.6 Da. The distribution of all the proteins identified according to the Protein Mass (kDa); the distribution of 20,453 peptides identified according to the length; and the distribution of 2717 proteins identified according to the sequence coverage were determined.

### 4.8. Differentially Expressed Analysis

A total of 2717 proteins were identified for this project, with (C-)/TMT and **MTX**/TMT used to determine the protein differential expression between the MTX/TMT and **IA**/TMT treatments and establish the following ratios: MTX/C, and **IA**/C, **IA**/MTX. The screening criteria used for the relative quantitation of proteins were fold-change > 1.5 (upregulated) or <0.67 (downregulated); each comparison was analyzed using cell signaling pathways and enrichment process analysis.

### 4.9. Bioinformatic Methodology

Since the raw data or the fold change cutoff scale from >1.5 to <0.67 in continuous values cannot be used in the analysis process due to software requirements, the ratios are not symmetrical around one. Therefore, the log ratio (Log1.5) is the log of the fold changes, i.e., log1.5 (condition1/condition2). Log ratios are used/plotted in graphs for the exploratory analysis of pathways and the functional enrichment of the cellular processes in each of the different treatments, generating a log ratio value matrix with each protein; these are better to show because they center around 0, giving reductions a negative value and increments a positive value, and because the log ratios are symmetrical around zero. The log ratio data matrix was used to feed the algorithms for the analysis of cellular process enrichment networks and signaling pathways using programming language R v4.2.2 and Rstudio v3.1.4. Applying the clusterProfiler v4.9.0, MSigDB in R (Molecular Signatures Database, accessed 15 July 2024), we enriched the plot and ggplo2 packages (accessed 15 July 2024); we also used symbol and mouse ID data (org.Mm.eg.db, accessed 15 July 2024), as well as the KEGG databases (Kyoto Encyclopedia of Genes and Genomes, accessed 15 July 2024); Gene ontology (GO), Biological Process (BP), Molecular Function (MF), and Cellular Component (CC) databases; and Reactome database. The enrichKEGG, enrichGO and compareCluster functions were applied, as indicated in the developer’s manuals. The results were plotted with the functions “emapplot”, “aplot_list”, “dotplot” and “cnetplot” for visualization [58].

### 4.10. Comparison of Shared Processes and Molecules

Proteins with differential changes between treatments were explored with the help of the G:Profiler tool that is available online; graphs and pathway enrichment tables were generated by comparing the proteins with the KEGG, GO and REACTOME databases [59]. The enrichment plot images generated in the *X*-axis show the number of different enriched processes according to each database, while the *Y*-axis plots the significance value obtained in ascending order (−log10 adj-*p*-value); a dashed cut-off at the top of the graph divides the most significant enriched cellular processes, while a black circle highlights a number of different processes of interest according to each database, according to the order in which they appear in a general list of enriched processes. At the bottom is a list of representative processes arranged by the significance value, with the first having the highest value. The list is labeled by ID (number in the general list of the process), resource (database with which the process was enriched), term ID (identification of the process according to each platform), term name (name of the enriched process according to each database) and adj-P-value (significance value of the process), shown with the number and color scale and arranged in descending order. Finally, to determine how many molecules and cellular processes are shared between the different conditions, the modulated protein data and their change values were analyzed using the DiVenn v2.0 online tool, as indicated by the developer [60]; in addition, Venn diagrams of the protein list were generated using the Venny v2.1 platform, following the instructions available for the manual tool [61]. All annotations referring to the proteins are based on the UNIQID gene name or UNIPROT-ID, found at http://geneontology.org/; https://reactome.org/; https://www.uniprot.org/ or https://www.genome.jp/kegg/pathway.html (accessed 15 July 2024).

### 4.11. In Silico Studies, Molecular Docking

The chemical structure of ligand methotrexate (CID: 126941) was retrieved from the chemical library PubChem (https://pubchem.ncbi.nlm.nih.gov/) (accessed on 10 October 2024). The chemical structure of the ligand incomptine-A was drawn, optimized, and submitted for energetic and geometrical minimization using Avogadro software [62]. The target enzymes were chosen by first considering which enzymes were altered by incomptine A, by secondly considering which crystal structure would have been reported in Protein Data Bank, and by thirdly considering that the results of PPI suggest that these were the proteins with the most important activity in our study. The proteins ADP/ATP translocase (P48962), alpha actin 3 (O88990), myozenin-1 (Q9JK37), 28S ribosomal protein S22 (Q9CXW2), actin alpha skeletal muscle (4b1u), alpha-actin-2 (Q9JI91), beta enolase (2xsx), gamma enolase (4zcw), histone H2AX (5gt0), histone H3.3 (5xm0), histone H4 (1f66), methyl-CPG-binding protein 2 (7mwk), myosin light chain 3 (5tby), myosin regulatory light chain 2 [ventricular/cardiac muscle isoform] (8efd), myosin regulatory light chain 2 [skeletal muscle isoform] (P97457), myosin-1 (Q5SX40), myosin-3 (P13541), myosin-4 (Q5SX39), myosin-7 (Q91Z83), myosin-8 (P13542), myosin-binding protein C fast type (1x5y), Dihydrofolate reductase (P00375), and monofunctional C1-tetrahydrofolate synthase (Q3V3R1) were selected for the in silico study. These were retrieved from the Protein Data Bank (http://www.rcsb.org, accessed 15 July 2024), accessed on 10 October 2024. The proteins were optimized for the molecular docking study; thus, all water molecules and ions were stripped, preserving the entire protein structure. Then, polar hydrogen atoms were added and ionized in a basic environment (pH = 7.4). Finally, Gasteiger charges were added.

Blind docking was carried out in all proteins with the aim of determining the possible binding site of the ligands proposed; considering the above, the molecular docking studies were performed using Autodock 4.2 software [63], and the search parameters used in all proteins were as follows: a grid-based procedure was used to create affinity maps with a grid box of 126 × 126 × 126 Å^3^ and a grid point spacing of 0.375 Å; the grid centers (x, y, z) for each protein search were −2.423, 2.685 and −2.649 for ADP/ATP translocase (P48962), −6.473, 2.387 and 9.077 for alpha actin 3 (O88990), −2.842, −1.66 and −4.098 for myozenin-1 (Q9JK37), −2.799, 2.87 and −1.94 for 28S ribosomal protein S22 (Q9CXW2), 28.883, −15.753 and 10.031 for actin alpha skeletal muscle (4b1u), −6.309, 1.745 and 9.678 for alpha-actin-2 (Q9JI91), −16.559, 4.064 and −3.963 for beta enolase (2xsx), 78.558, 113.313 and −87.573 for gamma enolase (4zcw), 18.124, 21.962 and 33.539 for histone H2AX (5gt0), 20.708, 22.683 and 57.869 for histone H3.3 (5xm0), 7.724, −3.726 and 4.202 for histone H4 (1f66), 31.763, 15.496 and −17.791 for methyl-CPG-binding protein 2 (7mwk), 58.178, 97.831 and 2.38 for myosin light chain 3 (5tby), 300.988, 275.348 and 236.601 for myosin regulatory light chain 2 [ventricular/cardiac muscle isoform] (8efd), −6.258, −0.436 and 0.774 for myosin regulatory light chain 2 [skeletal muscle isoform] (P97457), −12.807, 8.847 and −3.099 for myosin-1 (Q5SX40), −8.24, 4.234 and 13.231 for myosin-3 (P13541), −12.031, −2.854 and −6.154 for myosin-4 (Q5SX39), −12.681, 2.321 and −9.691 for myosin-7 (Q91Z83), −10.464, 10.528 and −6.055 for myosin-8 (P13542), 1.853, −4.695 and −0.625 for myosin-binding protein C fast type (1x5y), 12.283, −3.619 and −10.43 for dihydrofolate reductase (P00375), and −0.879, 3.064 and 9.938 for monofunctional C1-tetrahydrofolate synthase (Q3V3R1). The Lamarckian algorithm was employed, with an initial population of 100 individuals and a maximum number of energy evaluations of 1 × 10^7^ cycles. The interactions between enzyme and ligands were visualized with BIOVIA software (BIOVIA, Dassault Systems, v24.11.0.23298, San Diego, CA, USA).

Molecular docking studies were performed using Autodock 4.2 software [63], and the search parameters used in all proteins were as follows: a grid-based procedure was used to create affinity maps with a grid box of 126 × 126 × 126 Å^3^ and a grid point spacing of 0.375 Å. The Lamarckian algorithm was employed with an initial population of 100 individuals and a maximum number of energy evaluations of 1 × 10^7^ cycles. Interactions between the enzyme and inhibitor were visualized with PyMOL software (PyMOL Molecular Graphics System, Ver 2.0, Schrödinger, LLC, DeLano Scientific, San Carlos, CA, USA).

## 5. Conclusions

Our study, supporting the use of five approaches, including TMT, gene ontology (GO), Reactome, KEGG pathway, and molecular docking analyses, suggests that DEPs, Myh3, Eno2, and H4c11 may be three novel biomarkers or therapeutic targets for non-Hodgkin lymphoma. Also, we found that incomptine A has antitumor potential as un agent for the treatment of NHL. Bioinformatics techniques including GO, Reactome, KEGG pathway, and molecular docking analyses may be used as tools to identify the potential targets of drugs in NHL. In addition, this work could be a starting point for subsequent research, such as clinical trials. Also, Myh3, Eno2, and H4c11 are proteins that need further exploration to validate their participation in NHL. Finally, this work showed that **IA** is a compound with multiple targets and multiple pathways.

## Figures and Tables

**Table 1:** Anti-lymphoma activity of dichloromethane extract from the leaves of *D. incompta*, incomptine A (**IA**), and methotrexate (**MTX**).

Sample	Anti-Lymphoma Activity
	EC_50_ mg/kg (μmol/kg) in Male Balb/c Mice *
DCMDi	80.0 ± 1.4
Incomptine A (**IA**)	7.8 ± 0.02 (16.42)
Methotrexate (**MTX**)	1.9 ± 0.01 (2.75)

* r^2^ > 0.9800; results are expressed as average ± S.E.M (*n* = 6), *p* < 0.05; DCMDi, dichloromethane extract from the leaves of *D. incompta*.

**Table 2:** Differentially expressed proteins, from 2717 proteins identified according to their quantitation compared to the negative control.

C-(DMSO) vs.	Downregulated FC < 0.67 (<1/1.5)	Upregulated FC > 1.5
**MTX**	111	63
**IA**	193	141

Data represent the fold change (FC) divided into two categories with the number of differentially expressed proteins. Negative control (C-) versus treatments with differentially expressed proteins. No treatment and the negative control only inoculated with U-937 cells (C-), methotrexate (**MTX**) at a dose of 1.25 mg/kg, and incomptine A (**IA**) at a dose of 5 mg/kg. Differentially expressed proteins were compared against mouse protein.

**Table 3:** Protein dysregulation induced only by **IA** (5 mg/kg) in lymph nodes from male Balb/c mice with NHL and treated with **IA** or **MTX** (1.25 mg/kg) and identified by TMT.

Protein ID	Protein Name	Gene Name	IA (FC)	MTX (FC)
P49717	DNA replication licensing factor MCM4	Mcm4	0.50837903	WC
Q64339	Ubiquitin-like protein ISG15	Isg15	0.60919551	WC
Q8CI51	PDZ and LIM domain protein 5	Pdlim5	0.6338016	WC
Q9QXV3	Inhibitor of growth protein 1	Ing1	0.64274579	WC
Q9WVR4	RNA-binding protein	Fxr2	0.65477352	WC
Q80UW8	DNA-directed RNA polymerases I, II, and III subunit RPABC1	Polr2e	1.5312863	WC
P97315	Cysteine and glycine-rich protein 1	Csrp1	1.60834657	WC
Q9D0A3	Arpin	Arpin	1.62984839	WC
Q8BTS4	Nuclear pore complex protein Nup54	Nup54	1.63055079	WC
Q9Z2D6	Methyl-CpG-binding protein 2	Mecp2	1.77536209	WC
O70494	Transcription factor Sp3	Sp3	1.97910072	WC
P27661	Histone H2AX; Histone H2A type 1-A	H2afx	2.1980563	WC
P84244	Histone H3.3; Histone H3.3C	H3f3a	2.25523584	WC
P62806	Histone H4	H4c11 or Hist1h4a	2.79300107	WC

FC, fold change; WC, without change.

**Table 4:** Protein dysregulation induced by **IA** and **MTX** in lymph nodes from male Balb/c mice with NHL and treated with **IA** (5 mg/kg) or **MTX** (1.25 mg/kg) and identified by TMT.

Protein ID	Protein Name	Gene Name	IA (FC)	MTX (FC)
O09165	Calsequestrin-1	Casq1	0.12888545	0.16602143
O70250	Phosphoglycerate mutase 2	Pgam2	0.13830206	0.15385818
P13412	Troponin I, fast skeletal muscle	Tnni2	0.14277684	0.15563201
P20801	Troponin C, skeletal muscle	Tnnc2	0.15714623	0.17833863
P97457	Myosin regulatory light chain 11	Myl11	0.16594298	0.18569035
P17183	Gamma-enolase	Eno2	0.16642046	0.20448029
P13542	Myosin-8	Myh8	0.1686355	0.18800321
Q9QZ47	Troponin T, fast skeletal muscle	Tnnt3	0.1731853	0.18766126
P58774	Tropomyosin beta chain	Tpm2	0.17536152	0.19729003
P32848	Parvalbumin alpha	Pvalb	0.17847149	0.19727712
Q5SX39	Myosin-4	Myh4	0.18171385	0.19169617
P58771	Tropomyosin alpha-1 chain	Tpm1	0.18568101	0.21546771
Q9WUZ7	SH3 domain-binding glutamic acid-rich protein	Sh3bgr	0.1934887	0.21427406
P05977	Myosin light chain 1/3, skeletal muscle isoform	Myl1	0.19581059	0.22374912
P07310	Creatine kinase M-type	Ckm	0.20829073	0.21826908
Q5SX40	Myosin-1	Myh1	0.2083523	0.22498888
Q9JK37	Myozenin-1	Myoz1	0.22521654	0.24013324
P09542	Myosin light chain 3	Myl3	0.226636	0.21151913
Q9JKS4	LIM domain-binding protein 3	Ldb3	0.23224947	0.24548982
P13541	Myosin-3	Myh3	0.23853213	0.25998992
Q6P8J7	Creatine kinase S-type, mitochondrial	Ckmt2	0.2415395	0.26543409
Q8R429	Sarcoplasmic/endoplasmic reticulum calcium ATPase 1	Atp2a1	0.24265637	0.24623615
Q62234	Myomesin-1	Myom1	0.24575524	0.25292963
Q9WUB3	Glycogen phosphorylase, muscle form	Pygm	0.25940027	0.28057796
P23927	Alpha-crystallin B chain	Cryab	0.26406889	0.30707191
P51667	Myosin regulatory light chain 2, ventricular/cardiac muscle isoform	Myl2	0.26759405	0.2924342
Q5XKE0	Myosin-binding protein C, fast-type	Mybpc2	0.27308659	0.27462539
Q8R1X6	Spartin	Spg20	0.28107683	0.30937193
A2ASS6	Titin	Ttn	0.28504571	0.31076004
P04247	Myoglobin	Mb	0.28507773	0.31483925
P21550	Beta-enolase	Eno3	0.30736135	0.30165768
Q9JI91	Alpha-actinin-2	Actn2	0.31713038	0.34973394
Q9R0Y5	Adenylate kinase isoenzyme 1	Ak1	0.33109887	0.34217395
O88990	Alpha-actinin-3	Actn3	0.33172374	0.33547715
P68134	Actin, alpha skeletal muscle	Acta1	0.33828177	0.35181042
Q9JLH8	Tropomodulin-4	Tmod4	0.35491287	0.41950757
P48962	ADP/ATP translocase 1	Slc25a4	0.35701493	0.29200636
P97447	Four and a half LIM domains protein 1	Fhl1	0.36193865	0.3850256
Q9DAZ9	Abscission/NoCut checkpoint regulator	Zfyve19	0.36342725	0.42429163
Q9JIF9	Myotilin	Myot	0.37643825	0.44266943
P11404	Fatty acid-binding protein, heart	Fabp3	0.3966988	0.50535282
Q3TJD7	PDZ and LIM domain protein 7	Pdlim7	0.42645618	0.58178029
A2ABU4	Myomesin-3	Myom3	0.43217423	0.50573745
Q7TQ48	Sarcalumenin	Srl	0.43931245	0.4520274
Q8BV66	Interferon-induced protein 44	Ifi44	0.44047282	0.48108554
P04919	Band 3 anion transport protein	Slc4a1	0.44113319	0.39039966
Q91Z83	Myosin-7	Myh7	0.45691314	0.43288348
Q64345	Interferon-induced protein with tetratricopeptide repeats 3	Ifit3	0.46633725	0.50007841
Q8VHX6	Filamin-C	Flnc	0.50523832	0.52866406
Q9CQC7	NADH dehydrogenase [ubiquinone] 1 beta subcomplex subunit 4	Ndufb4	0.51206722	0.45982722
P15307	Proto-oncogene c-Rel	Rel	0.56283075	0.5481155
P45591	Cofilin-2	Cfl2	0.59974682	0.62451683
Q9D1L0	Coiled-coil-helix-coiled-coil-helix domain-containing protein 2	Chchd2	0.6129121	0.63553402
Q99NB8	Ubiquilin-4	Ubqln4	0.61305215	0.57611773
Q9CXW2	28S ribosomal protein S22, mitochondrial	Mrps22	0.61952001	0.60291528
Q9DA08	SAGA-associated factor 29 homolog	Ccdc101	0.62412377	0.65610408
Q3V3R1	Monofunctional C1-tetrahydrofolate synthase, mitochondrial	Mthfd1l	0.7976781	0.7363416
Q501J7	Phosphatase and actin regulator 4	Phactr4	1.62538381	1.50598278
P26645	Myristoylated alanine-rich C-kinase substrate	Marcks	2.41370534	1.67943824
P00375	Dihydrofolate reductase	Dhfr	1.675288	1.5253043

FC, fold change.

**Table 5:** ∆G (kcal/mol), Ki (µM), and receptor–ligand interactions from molecular docking.

Protein	Ligands							
	Incomtine A				Methotrexate (M)			
	ΔG	Ki	H-BR	NPI	ΔG	Ki	H-BR	NPI
ADP/ATP translocase	−4.35	649.2	Pro28, Ile29, Val32, Phe67, Phe70, Trp71	Val58	−5.24	144.2	Gln175, Gly176, Phe177, Ser178, Met238, Thr254	Cys129, Leu170, Lys171, Tyr174
Alpha-actin 3	−4.72	344.3	Arg305, Thr306, Asp328, His336	Trp302, Trp309, Tyr332	−4.87	269.4	Arg333, Arg334, Leu335, Pro338, Val341, Gln342, Lys695, Glu 702, Met 723, Arg727, Trp730, Glu731	Pro339, Leu734
Myozenin-1	−4.7	356.4	Val44, Pro263, Ser264, Phe265, Thr268	Arg267, Pro269, Ile270, Trp272	−3.83	1560	Phe70, Glu73, Asn74, Ser82, Ser83, Met84	His86, Phe87, Phe90, Pro92
28S ribosomal protein S22	−4.82	291.5	Met70, Gln75, Arg76, Thr79, Val123	Lys122, Leu125, Lys126, Met127	−4.95	235.5	Lys126, Met127, Lys198, Arg201, Phe234, Glu235, Tyr241, Arg265	Pro128, Pro129, Val231, Pro236
Actin alpha skeletal muscle	−4.79	309.4	Glu107, Ala108, pro109, Leu110, Asn111, Arg116, Ala135, Tyr169, Ile175, Cys374, Phe375	Ile136, Leu159, Ala170, Pro172	−5.73	63.4	Gly15, Tyr69, Asp157, Arg183, Glu207, Arg210, Glu214	Leu16, Val30, Tyr337
Alpha-actin-2	−4.69	362.8	Trp296, Arg299, Thr300, Asp322, Asp325, Tyr326, His330	Trp303	−6.12	32.7	Arg328, Pro333, Gln336, Cys339, Lys689, Trp724, Glu725	Lys329, His330, Pro332, Val335, Leu728
Beta-enolase	−6.12	32.7	Arg15, Ser37, gly38, Ala39, Ser40, Thr41, Glu48	Arg50, Lys60	−7.31	4.37	Asp13, Asn17, Pro18, Gly38, Ala39, Ser40, Glu48, Arg50, Gly52, Leu58, Gly59, Gly374	Arg15, Lys54, Lys60
Gamma enolase	−5.08	189.8	Gly174, Ala175, Glu176, ala421, Arg422, Phe423, Ala424, Gly425	Leu434	−6.47	17.9	Val173, Gly174, ala175, Glu176, Ser177, Ala421, Arg422, Phe423, Ala424, Gly425, His426, Asn427, Leu434	-
Histone H2AX	−4.25	762.1	Leu34, Leu51, Leu55	Ile30, Leu33, Tyr39	−4.23	794.7	Tyr57, Leu58, Glu61, Leu65, Asp90, Leu93, Lys95	Glu92, Leu96
Histone H3.3	−4.47	528.1	Glu50, Ile51, Tyr54, Gln55, Leu61	Val101, Phe 104	−3.34	3590	Ser96, Tyr99, Leu100, Arg128, Glu133	Ile62, Phe67, Leu70, Arg128
Histone H4	−4.71	354.2	Asp224, Asn225, Ile226, Ile229, Leu258, Glu263, Ile266	Lys259, Leu262	−2.12	2813	Val257, Val265, Val286, Leu290, Leu297	Leu237, Leu258, Phe261, Leu262, Arg295
Methyl-CPG-binding protein 2	−5.33	123	Cys152, Pro153, ala 154, Leu155, Pro156, Leu197, Thr200, Val201	Pro157, Tyr196	−4.58	440	Arg166, Asp176, Val177, Tyr178, Lys188, Ser189, Gln192, Phe208	-
Myosin light chain 3	−5.22	149.3	Gly136, Val148, Leu157, Val156, Leu160, Phe188, Ile192	Phe133, Leu137, Phe140, Leu153	−4.08	1002	Gly84, Gln85, Ile121, Ser122, Lys123, Asn124, Lys125, Thr127	Ile44, Asn86
Myosin regulatory light chain 2 Ventricular/cardiac muscle isoform	−5.1	182.4	Asn126, Tyr128, Lys129, Trp130, Tyr134, Asn187, Arg190	Leu131, Val133, Val186	−5.09	182.2	Ile467, Phe468, Asp469, Arg567, Val586, Asp587	Asn471, Ile569, Lys572
Myosin regulatory light chain 2 Skeletal muscle isoform	−5.72	64.3	Ile45, Met71, ala74, Ile78, Phe83, Lys90	Ile37, Leu50, Met70, Met86	−4.06	1006	Ser20, Met21, Phe22, Gln26, Phe87, Gly88, Glu89, Leu91, Gly93	Lys92
Myosin-1	−4.42	579.8	Trp441, Thr444	Lys437, Leu440, Leu623, Ty622	−5.23	147	His495, Glu500, Glu502, Glu503	Leu499, Lys505, Lys506, Arg719
Myosin-3 (Myh3)	−5.35	120.5	Thr628, Asp629, Lys634, Lys635, Lys636, Val637, Ala638, Lys639, Lys640, Lys641, Ser643	-	−4.42	578.6	Lys635, Val637, Glu895, Leu909, Lys1618, Asp1622	Lys634, Lys636, Ala638, Leu899, Ala902, Cys906, Met1619
Myosin-4	−4.2	840.3	Gln490, Phe491, His494, Ala521, Ile524, Glu525, Asn660, Met663, Lys667	Lys487	−5.65	72.3	Leu121, His495, Met496, Glu500, Glu502, Glu503, Pro671	Leu499, Arg716
Myosin-7	−3.97	1240	Thr167, Asp168, Glu170, Gln172, Pro710, Asn711, Arg712, Leu714, Arg721, Leu770	Tyr164, His666, Ile713	−4.68	225.4	Glu497, Glu499, Glu500, Lys503, Gly505	Leu496, Lys502, His760
Myosin-8	−6.09	34.4	Thr268, Tyr269, Leu270, Leu271, Lys273, Glu436, Phe439, Phe654, Asn657	Leu440, Val650, Leu653	−4.36	639.3	Glu500, Glu502, Glu503, Lys505	Leu499, Lys506, Arg715
Myosin-binding protein C, fast-type	−4.26	760.3	Pro57, Ala58, Asn59, Phe68, Val70, Lys71, Asp72	Tyr47, Val56, Lys60	−4.23	795.1	Thr16, Glu18, Asp19, Leu98, Gln99, Pro100, Thr102, Arg104, Glu105, Ser106, Gly107	Val20, Leu97, Val101
Dihydrofolate reductase	−8.02	13.2	Ala10, Asp22, Leu23, Trp25, Glu31, Phe32, Thr57, Ser60, Pro62, Val122	Val9, Ile17, Phe35, Ile61, Leu68	−8.92	12.8	Val9, Glu31, Tyr34, Gln36, Thr57, Ser60, Pro62, Asn65, Leu68, Arg71, Val116, Tyr122, Phe115, Thr137	Ile8, Ala10, Leu23, Phe32, Phe35, Ile61
Monofunctional C1-tetrahydrofolate synthase	−6.95	8.06	Asn147, Glu148, Pro150, Lys173, Glu175, Lys176, Trp336, Glu339, Gln340, Ala384, Asp385	Leu383	−6.02	38.3	Gln341, His342, Arg344, Glu403, Arg404, Leu405, Lys406, Asp407, Gln408, Asn446, Val701, Gly702, Glu703, Glu704, Gly705	His342, Asp407

ΔG: Binding energy (kcal/mol^−1^); H-BR: H-binding residues; NPI: Non-polar interactions; Asp: Aspartate; Asn: Asparagine; Arg: Arginine; Gln: Glutamine; Lys: Lysine; Thr: Threonine; Ser: Serine; Trp: Tryptophan; Leu: Leucine; His: Histidine; Gly: Glycine; Glu: Glutamic acid; Ile: Isoleucine; Tyr: Tyrosine; Phe: Phenylalanine.

## Data Availability

Data of this study is available for request from corresponding author.

## Supplementary Materials

The following supporting information can be downloaded at: https://www.mdpi.com/article/10.3390/ph18010005/s1, Figure S1. Results of molecular docking on proteins, 2D representation of interactions between Incomptine A (**1**) and Methotrexate (**2**) in (**A**) ADP/ATP translocase, (**B**) Alpha-actin3, (**C**) Myozenin-1, (**D**) 28S ribosomal protein S22, (**E**) Actin, alpha skeletal muscle, (**F**) Alpha-actin-2, (**G**) Beta-enolase, (**H**) Gamma enolase (Eno2), (**I**) Histone H2AX, (**J**) Histone H3.3, (**K**) Histone H4 (Hist1h4a or H4c11), (**L**) Methyl-CPG-binding protein 2, (**M**) Myosin light chain 3, (**N**) Myosin regulatory light chain 2, ventricular/cardiac muscle isoform **O)** Myosin regulatory light chain 11 (**P**) Myosin-1, (**Q**) Myosin-3 (Myh-3), (**R**) Myosin-4, (**S**) Myosin-7, (**T**) Myosin-8, (**U**) Myosin-binding protein C, fast-type, (**V**) Dihydrofolate reductase, and (**W**) Monofunctional C1-tetrahydrofolate dehydrogenase/cyclohydrolase.

## References

[1] Soerjomataram I., Bray F. (2021). Planning for tomorrow: Global cancer incidence and the role of prevention 2020–2070. Nat. Rev. Clin. Oncol..

[2] Sung H., Ferlay J., Siegel R.L., Laversanne M., Soerjomataram I., Jemal A., Bray F. (2021). Global cancer statistics 2020: GLOBOCAN estimates of incidence and mortality worldwide for 36 cancers in 185 countries. CA Cancer J. Clin..

[3] Hernandez-Ruiz E., Alvarado-Ibarra M., Juan Lien-Chang L.E., Banda-Garcia L., Aquino-Salgado J.L., Barragan-Ibanez G., Ramirez-Romero E.F., Nolasco-Cancino C., Herrera-Olivares W., Morales-Adrian J.J. (2021). Epidemiology and clinical characteristics of non-Hodgkin lymphoma in Mexico. World J. Oncol..

[4] Palacio-Mejia L.S., Hernandez-Avila J.E., Hernandez-Avila M., Dyer-Leal D., Barranco A., Quezada-Sanchez A.D., Alvarez-Aceves M., Cortes-Alcala R., Fernandez-Wheatley J.L., Ordonez-Hernandez I. (2022). Leading causes of excess mortality in Mexico during the COVID-19 pandemic 2020-2021: A death certificates study in a middle-income country. Lancet Reg. Health Am..

[5] Calzada F., Bautista E., Hidalgo-Figueroa S., Garcia-Hernandez N., Barbosa E., Velazquez C., Ordonez-Razo R.M., Arietta-Garcia A.G. (2021). Antilymphoma effect of incomptine A: In vivo, in silico, and toxicological studies. Molecules.

[6] INEGI Estadísticas de Mortalidad/Defunciones Registradas (Mortalidad General). https://www.inegi.org.mx/programas/mortalidad/#Tabulados.

[7] Howard S.C., McCormick J., Pui C.H., Buddington R.K., Harvey R.D. (2016). Preventing and managing toxicities of high-dose methotrexate. Oncologist.

[8] Narkhede M., Yazdy M.S., Cheson B.D. (2019). Targeting biology in non-Hodgkin lymphoma. Hematol. Oncol. Clin. N. Am..

[9] Valla K., Flowers C.R., Koff J.L. (2018). Targeting the B cell receptor pathway in non-Hodgkin lymphoma. Expert Opin. Investig. Drugs.

[10] Babaei G., Aliarab A., Abroon S., Rasmi Y., Aziz S.G. (2018). Application of sesquiterpene lactone: A new promising way for cancer therapy based on anticancer activity. Biomed. Pharmacother..

[11] Pina-Jimenez E., Calzada F., Bautista E., Ordonez-Razo R.M., Velazquez C., Barbosa E., Garcia-Hernandez N. (2021). Incomptine A induces apoptosis, ROS production and a differential protein expression on non-Hodgkin’s lymphoma cells. Int. J. Mol. Sci..

[12] Calzada F., Garcia-Hernandez N., Hidalgo-Figueroa S., Bautista E., Barbosa E., Velazquez C., Hernandez-Caballero M.E. (2022). Expanding the study of the cytotoxicity of incomptines A and B against leukemia cells. Molecules.

[13] Rauniyar N., Yates J.R. (2014). Isobaric labeling-based relative quantification in shotgun proteomics. J. Proteome Res..

[14] Zhang L., Elias J.E. (2017). Relative protein quantification using tandem mass tag mass spectrometry. Methods Mol. Biol..

[15] Bautista E., Calzada F., Yepez-Mulia L., Chavez-Soto M., Ortega A. (2012). Incomptines C and D, two heliangolides from *Decachaeta incompta* and their antiprotozoal activity. Planta Medica.

[16] Seshi B. (2008). An integrated approach to mapping the protome of the human bone marrow stromall cel. Proteomics.

[17] Zhang H., Lv L., Liu H., Cui L., Chen G., Bi P., Li Z. (2009). Profiling the potential biomarkers for cell diferentiation of pancreatic cancer using iTRAQ and 2-D LC-MS/MS. Proteom. Clin. Appl..

[18] Muraoka S., Kume H., Watanabe S., Adachi J., Kumano M., Sato M., Kawasaki N., Kodera Y., Ishitobi M., Inaji H. (2012). Strategy for SRM-based verification of biomarker candidates discovered by iTRAQ method in limited breast cancer tissue samples. J. Proteome Res..

[19] Chen J.S., Chen K.T., Fan C.W., Han C.L., Chen Y.J., Yu J.S., Chang Y.S., Chien C.W., Wu C.P., Hung R.P. (2010). Comparison of membrane fraction proteomic profiles of normal and cancerous human colorectal tissues with gel-assisted digestion and ITRAQ labeling mass spectrometry. FEBS J..

[20] Zhang Z., Zhang L., Hua Y., Jia X., Li J., Hu S., Peng X., Yang P., Sun M., Ma F. (2010). Comparative proteomic analysis of plasma membrane proteins between human osteosarcoma and normal osteoblastic cell lines. BMC Cancer.

[21] Kolberg L., Raudvere U., Adler P., Vilo J., Peterson H. (2023). g:Profiler-interoperable web service for functional enrichment analysis and gene identifier mapping (2023 update). Nucleic Acids Res..

[22] Han R., Yoon H., Kim G., Lee H., Lee Y. (2023). Revolutionizing medicinal chemistry: The application of artificial intelligence (AI) in early drug discovery. Pharmaceuticals.

[23] Zhang D.H., Wu K.L., Zhang X., Deng S.Q., Peng B. (2020). In silico screening of Chinese herbal medicines with the potential directly inhibit 2019 novel coronavirus. J. Integr. Med..

[24] Amparo T.R., Seibert J.B., Almeida T.C., Costa F.S.F., Silveira B.M., da Silva G.N., dos Santos O.D.H., Souza G.H.B. (2020). In silico approach of secondary metabolites from Brazilian herbal medicines to search for potential drugs against SARS-CoV-2. Phytother. Res..

[25] Global Burden of Disease Cancer Collaboration (2018). Global, Regional, and National Cancer Incidenece, Mortality, Years of Life Lost, Years Lived With Disability, and Disability-Adjusted Life-Years for 29 Cancer Groups, 1990 to 2016: A systematic analysis for Global Burden Disease Study. JAMA Oncol..

[26] Shankland K.R., Armitage J.O., Hancock B.W. (2012). Non-Hodgkin lymphoma. Lancet.

[27] Ramirez-Santos J., Calzada F., Mendieta-Wejebe J.E., Ordoñez_Razo R.M., Martinez-Casares R.M., Valdes M. (2022). Understanding the antilymphoma activity of *Annona macroprophyllata* Donn and its acyclic terpenoids: In vivo, in vitro, and in silico studies. Molecules.

[28] Escandón-Rivera S., González-Andrade M., Bye R., Linares E., Navarrete A., Mata R. (2012). a-glucosidase inhibitors from *Brickellia cavanillesii*. J. Nat. Prod..

[29] Zhang S., Won Y.K., Ong C.W., Shen H.M. (2005). Anti-cancer potential of sesquiterpene lactones: Bioactivity and molecular mecahnism. Curr. Med. Chem..

[30] Sepulveda-Robles O., Espinoza-Gutiérrez B., Gómez-Verjan J.C., Guzman-Gutierrez S.L., De Ita M., Silva-Miranda M., Espitia-Pinzón C.I., Fernández-Ramírez F., Herrera-Salazar A., Mata-Rocha M. (2019). Trypanocidal and toxicological assessment in vitro and in silico of three sesquiterpene lactones from Asteraceae plant species. Food Chem. Toxicol..

[31] Kreuger M.R.O., Grootjans S., Biavatti M.W., Vandenabeele P., D’Herde K. (2012). Sesquiterpene lactone as drugs with multiple targets in cancer treatment: Focus parthenolide. Wolter Klumer Health.

[32] Wen J., You K.R., Lee S.Y., Song C.H., Kim D.G. (2002). Oxidative stress.mediated apoptosis: The anticancer effect of the sesquiterpene lactones parthenolide. J. Biol. Chem..

[33] Mathema V.B., Koh Y.S., Thankuri B.C., Sillanpaa M. (2012). Parthenolide, a sesquiterpene lactone, expresses multiple anti-cancer and anti-inflammatory activities. Inflammation.

[34] Meng Y., Huang K., Shi M., Huo Y., HaN L., Liu B., Li Y. (2023). Research advances in the role of tropomyosin family in cancer. Int. J. Mol. Sci..

[35] Lo L.H., Lam C.Y., To J.C., Chiu C.H., Keng V.W. (2021). Sleeping beauty insertional mutagenesis screen identifies the pro-metastatic roles of CNPY2 and ACT2 in hepatocellular carcinoma tumor progression. Biochem. Biophys. Res. Commun..

[36] Li M., Sun Q., Wang X. (2017). Transcrptional landscape of human cancers. Oncotarget.

[37] Li C., Guan R., Li W., Wei D., Cao S., Chang F., Wei Q., Wei R., Chen L., Xu C. (2023). Analysis of myosin genes in HNSCC and identify MYL1 as a specific poor prognostic biomarker, promotes tumor metastasis and correlates with tumor immune infiltration in HNSCC. BMC Cancer.

[38] Chai Y., Liu X., Jiang Y., Zhou Y., Xiu W. (2024). TNNC2 is a microsatellite instability-related gene for response to immune checkpoint blocking therapy in colorectal cancer and contributes to tumor progression. J. Biol. Regul. Homeost. Agents.

[39] Fu Z., Liang X., Shi L., Tang L., Chen D., Liu A., Shao C. (2021). SYT8 promotes pancreatic cancer progression via the TNNI2/ERRa/SIRT1 signaling pathway. Cell Death Discov..

[40] Toydemir R.M., Rutherford A., Whitby F.G., Jorde L.B., Carey J.C., Bamshad M.J. (2006). Mutations in embryonic myosin heavy chain (MYH3) cause Freeman-Sheldon syndrome and Sheldon-Hall syndrome. Nat. Genet..

[41] Hu G., Yao H., Wei Z., Li L., Yu Z., Li J., Luo X., Guo Z. (2023). A bioinformatic approach to identify a disufidptosis-related gene signature for prognostic implication in colon adenocarcinoma. Sci. Rep..

[42] Lee D.-Y., Kang Y., Im N.-R., Kim B., Kwon T.-K., Jung K.-Y., Baek S.-K. (2021). Actin-associated gene expression is aassociated with early regional metastasis of tongue cancer. Laryngoscope.

[43] Yu X.-J., Yang M.-J., Zhou B., Wang G.-Z., Huang Y.-C., Wu L.-C., Cheng X., Wen Z.-S., Huang J.-Y., Zhang Y.-D. (2015). Characterization of somatic mutations in air pollution-related lung cancer. EBioMedicine.

[44] Sun J., Li S., Wang F., Fan C., Wang J. (2019). Idetification of key pathways and genes in PTEN mutation prostae cancer by bioinformatics analysis. BMC Med. Genet..

[45] Bashin M., Reinherz E.L., Reche P.A. (2006). Recognition and classification of histones using support vector machine. J. Comput. Biol..

[46] Pan D., Chen J., Feng C., Wu W., Wang Y., Tong J., Zhou D. (2019). Preferential localization of MUC1 glycoprotein in exosomes secreted by non-small cell lung carcinoma cells. Int. J. Mol. Sci..

[47] Zhang Q., Hu H., Chen S.-Y., Liu C.-J., Hu F.-F., Yu J., Wu Y., Guo A.-Y. (2019). Transcriptome and regulatory network analyses of CD-19-CART-T immunotherapy for B-ALL. Genom. Proteom. Bioinform..

[48] Yan G., Dai M., Poulet S., Wang N., Boudreault J., Daliah G., Ali S., Lebrun J.-J. (2023). Combined in vitro/in vivo genome-wide CRISPR screens in triple negative breast cancer identify cancer stemness regulators in paclitaxel resistence. Oncogenesis.

[49] Soh M.A., Garrett S.H., Somji S., Dunlevy J.R., Zhou X.D., Sens M.A., Bathula C.S., Allen C., Sens D.A. (2011). Arsenic, cadmiun and neuron specific enolase (ENO2, g-enolase) expression in breast cancer. Cancer Cell Int..

[50] Liu C.-C., Wang H., Wang W.-D., Wang L., Liu W.-J., Wang J.-H., Geng Q.-R. (2018). ENO2 promotes cell proliferation, glycolys, and glucocorticoid-resistance inn acute lymphoblastic leukemia. Cell Physiol. Biochem..

[51] Isgró M.A., Bottoni P., Scatena R. (2015). Neuron-specif enolase as a biomarker: Biochemical and clinical aspects. Advances in Cancer Biomarkers, Advances in Experimental Medicine and Biology.

[52] Gao L., Yang F., Tang D., Xu Z., Tang Y., Tang Y., Yang D., Sun D., Chen Z., Teng Y. (2023). Mediation of PKM2-dependent glycolytic and non-glycolytic pathways by ENO2 in head and neck cancer development. J. Exp. Clin. Cancer Res..

[53] Liu D., Mao Y., Chen C., Zhu F., Lu W., Ma H. (2020). Expression patterns and clinical significance of ENO2 in lung cancer: An analysis based on oncomine database. Ann. Tranl. Med..

[54] Yukimoto R., Nishida N., Hata T., Fujino S., Ogino T., Miyoshi N., Takahashi H., Uemura M., Satoh T., Hirofumi Y. (2021). Specific activation of glycolytic enzyme enolase 2 in BRAF V600E-mutated colorectal cancer. Cancer Sci..

[55] Lv C., Yu H., Wang K., Chen C., Tang J., Han F., Mai M., Ye K., Lai M., Zhang H. (2022). ENO2 promotes colorectal cancer metastasis by interacting with the LNCRNA CYTOR and activating YAP1-induced EMT. Cells.

[56] Chen Z., Li X.-Y., Guo P., Wang D.-L. (2021). MYBPC2 and MYL1 as significant gene markers for rhabdomyosarcoma. Technol. Cancer Res. Treat..

[57] (2001). Especificaciones Técnicas para la Producción, Cuidado y uso de Animales de Laboratorio.

[58] Wu T., Hu E., Xu S., Chen M., Guo P., Dai Z., Feng T., Zhou L., Tang W., Zhan L. (2021). clusterProfiler 4.0: A universal enrichment tool for interpreting omics data. Innovation.

[59] Raudvere U., Kolberg L., Kuzmin I., Arak T., Adler P., Peterson H., Vilo J. (2019). g:Profiler: A web server for functional enrichment analysis and conversions of gene lists (2019 update). Nucleic Acids Res..

[60] Sun L., Dong S., Ge Y., Fonseca J.P., Robinson Z.T., Mysore K.S., Mehta P. (2019). DiVenn: An Interactive and integrated web-based visualization tool for comparing gene lists. Front. Genet..

[61] Oliveros J.C. Venny. An Interactive Tool for Comparing Lists with Venn’s Diagrams. https://bioinfogp.cnb.csic.es/tools/venny/index.html.

[62] Hanwell M., Curtis D., Lonie D., Vandermeersch T., Zurek E., Hutchison G. (2012). Avogadro: An advanced semantic chemical editor, visualization, and analysis platform. J. Cheminform..

[63] Morris G., Lindstrom W., Sanner M., Belew R., Goodshell D., Olson A. (2009). Autodock4 and AutodockTools4: Automated docking with selective receptor flexibility. J. Comput. Chem..

