# Supplementary material for: Understanding the Molecular Mechanisms of Incomptine A in Treating Non-Hodgkin Lymphoma Associated with U-937 Cells: Bioinformatics Approaches, Part I
Source: Pharmaceuticals (Basel). 2024 Dec 24;18(1):5. doi: 10.3390/ph18010005 (PMC11768224; doi:10.3390/ph18010005)
Supplement: Supplementary file 1 [file pharmaceuticals-18-00005-s001.zip › pharmaceuticals-3318477-supplementary.pdf]

Understanding the Molecular Mechanisms of Incomptine A to Treat Non-Hodgkin Lymphoma Associated to U-937 cells: Bioinformatics Approaches, Part I

Fernando Calzada [1, *], Normand García-Hernández [2, *], Elihú Bautista [3], José Manuel Sánchez-López [1], Miguel Valdes[4], Claudia Velázquez[5], and Elizabeth Barbosa[4]

Supplementary material

Figure S1. Results of molecular docking on proteins, 2D representation of interactions between Incomptine A (**1**) and Methotrexate (**2**) in **A)** ADP/ATP translocase, **B)** Alpha-actin3, **C)** Myozenin-1, **D)** 28S ribosomal protein S22, **E)** Actin, alpha skeletal muscle, **F)** Alpha-actin-2 , **G)** Beta-enolase, **H)** Gamma enolase (Eno2), **I)** Histone H2AX, **J)** Histone H3.3, **K)** Histone H4 (Hist1h4a or H4c11), **L)** Methyl-CPG-binding protein 2, **M)** Myosin light chain 3, **N)** Myosin regulatory light chain 2, **O)** Myosin regulatory light chain 2 Skeletal muscle, **P)** Myosin-1, **Q)** Myosin-3 (Myh-3), **R)** Myosin-4, **S)** Myosin-7, **T)** Myosin-8 , **U)** Myosin-binding protein C, fast-type, **V)** Dihydrofolate reductase, and **W)** Monofunctional C1-tetrahydrofolate dehydrogenase/cyclohydrolase.

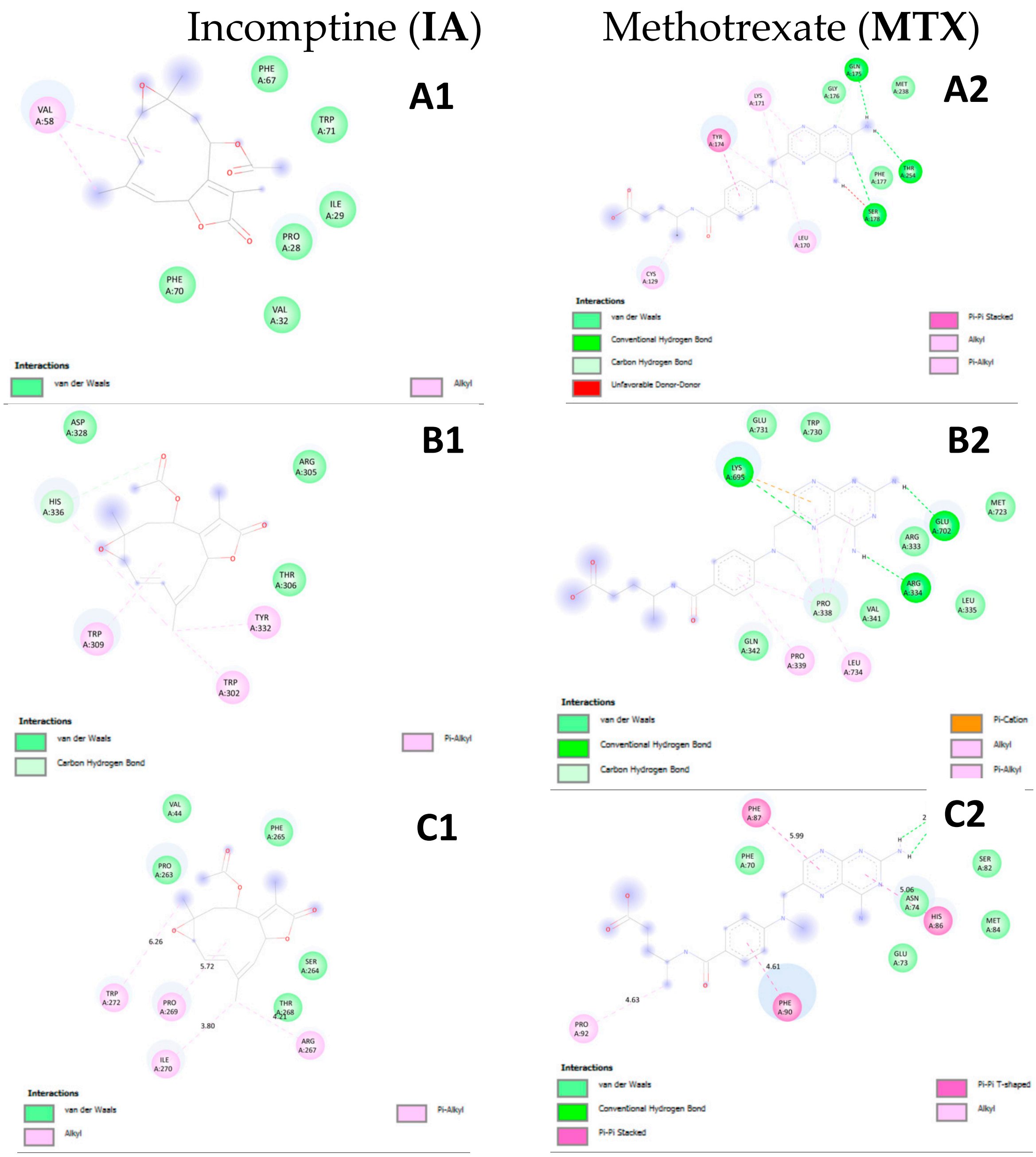

Incomptine (IA)
Methotrexate (MTX)
A1
PHE A:67
VAL A:58
TRP A:71
ILE A:29
PRO A:28
PHE A:70
VAL A:32
Interactions
van der Waals
Alkyl
A2
Interactions
van der Waals
Conventional Hydrogen Bond
Carbon Hydrogen Bond
Unfavorable Donor-Donor
Pi-Pi Stacked
Alkyl
Pi-Alkyl
B1
ASP A:328
ARG A:305
HIS A:336
THR A:306
TYR A:332
TRP A:309
TRP A:302
Interactions
van der Waals
Carbon Hydrogen Bond
Pi-Alkyl
B2
GLU A:731
TRP A:730
LYS A:695
MET A:723
GLU A:702
ARG A:333
ARG A:334
PRO A:338
VAL A:341
LEU A:335
GLN A:342
PRO A:339
LEU A:734
Interactions
van der Waals
Conventional Hydrogen Bond
Carbon Hydrogen Bond
Pi-Cation
Alkyl
Pi-Alkyl
C1
VAL A:44
PHE A:265
PRO A:263
6.26
5.72
SER A:264
TRP A:272
PRO A:269
THR A:268
3.80
ARG A:267
ILE A:270
Interactions
van der Waals
Alkyl
Pi-Alkyl
C2
PHE A:87
5.99
PHE A:70
SER A:82
5.06
ASN A:74
HIS A:86
MET A:84
GLU A:73
4.61
4.63
PHE A:90
PRO A:92
Interactions
van der Waals
Conventional Hydrogen Bond
Pi-Pi Stacked
Pi-Pi T-shaped
Alkyl

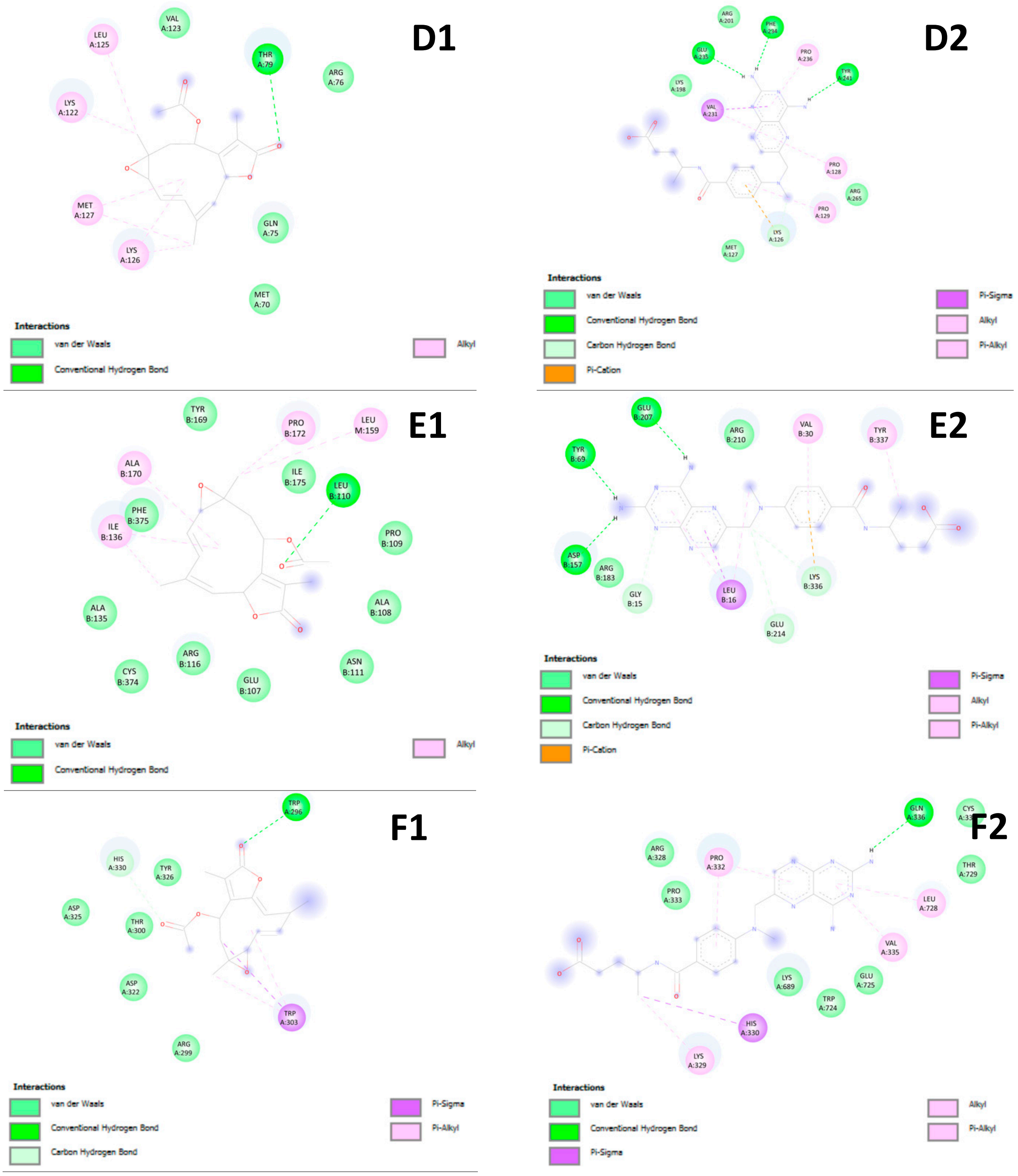
D1
LEU A:125
VAL A:123
THR A:79
ARG A:76
LYS A:122
MET A:127
LYS A:126
GLN A:75
MET A:70
Interactions
van der Waals
Conventional Hydrogen Bond
Alkyl
D2
Interactions
van der Waals
Conventional Hydrogen Bond
Carbon Hydrogen Bond
Pi-Cation
Pi-Sigma
Alkyl
Pi-Alkyl
E1
TYR B:169
PRO B:172
LEU M:159
ALA B:170
ILE B:175
LEU B:110
PHE B:375
ILE B:136
PRO B:109
ALA B:135
ALA B:108
ARG B:116
CYS B:374
GLU B:107
ASN B:111
Interactions
van der Waals
Conventional Hydrogen Bond
Alkyl
E2
GLU B:207
TYR B:69
ARG B:210
VAL B:30
TYR B:337
ASP B:157
ARG B:183
GLY B:15
LEU B:16
LYS B:336
GLU B:214
Interactions
van der Waals
Conventional Hydrogen Bond
Carbon Hydrogen Bond
Pi-Cation
Pi-Sigma
Alkyl
Pi-Alkyl
F1
TRP A:296
HIS A:330
TYR A:326
ASP A:325
THR A:300
ASP A:322
TRP A:303
ARG A:299
Interactions
van der Waals
Conventional Hydrogen Bond
Carbon Hydrogen Bond
Pi-Sigma
Pi-Alkyl
F2
GLN A:336
ARG A:328
PRO A:332
THR A:729
PRO A:333
LEU A:728
VAL A:335
LYS A:689
GLU A:725
TRP A:724
HIS A:330
LYS A:329
Interactions
van der Waals
Conventional Hydrogen Bond
Pi-Sigma
Alkyl
Pi-Alkyl

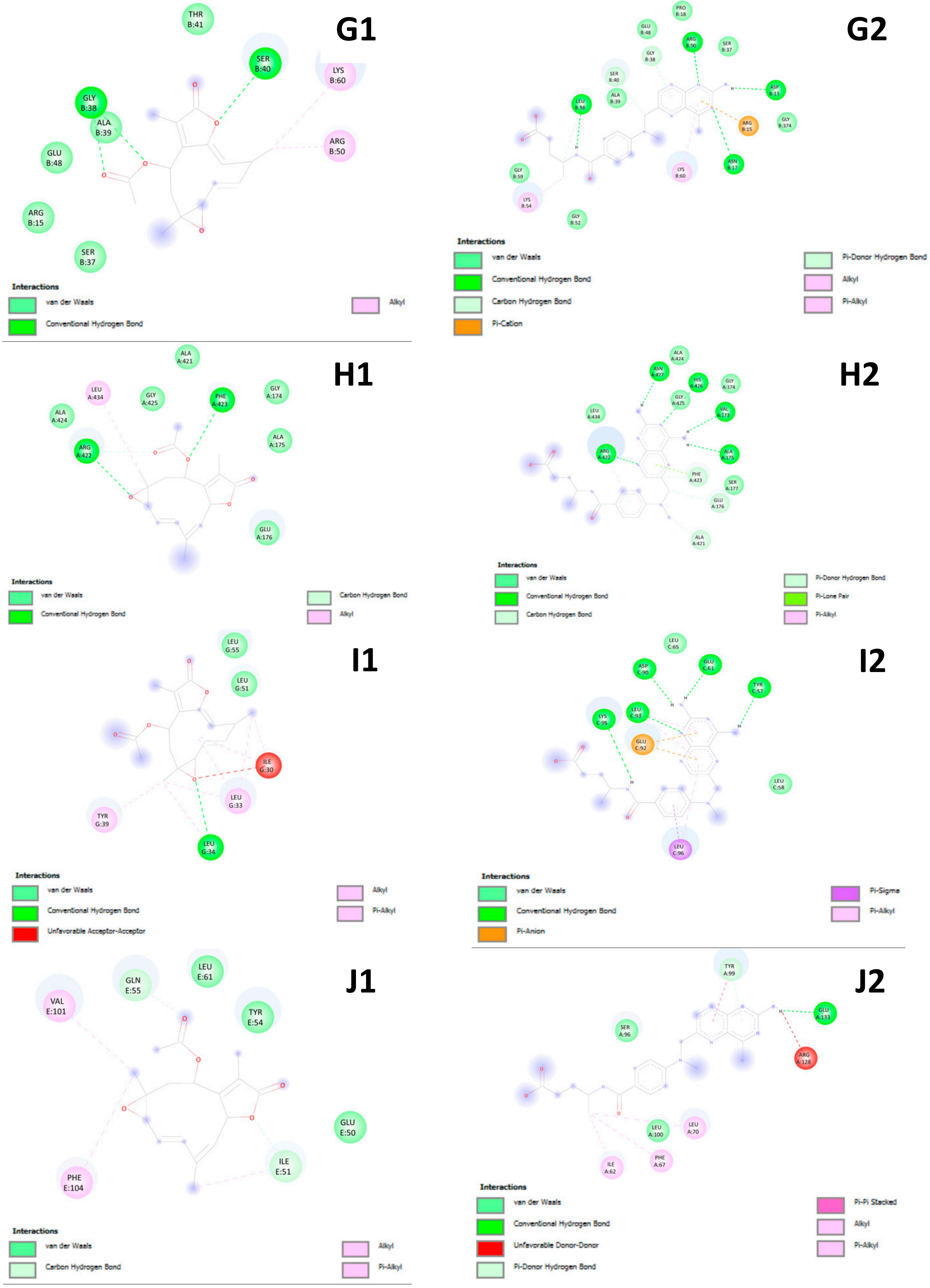
G1
THR B:41
SER B:40
LYS B:60
GLY B:38
ALA B:39
GLU B:48
ARG B:50
ARG B:15
SER B:37
Interactions
van der Waals
Conventional Hydrogen Bond
Alkyl
G2
Interactions
van der Waals
Conventional Hydrogen Bond
Carbon Hydrogen Bond
Pi-Cation
Pi-Donor Hydrogen Bond
Alkyl
Pi-Alkyl
H1
Interactions
van der Waals
Conventional Hydrogen Bond
Carbon Hydrogen Bond
Alkyl
H2
Interactions
van der Waals
Conventional Hydrogen Bond
Carbon Hydrogen Bond
Pi-Donor Hydrogen Bond
Pi-Lone Pair
Pi-Alkyl
I1
Interactions
van der Waals
Conventional Hydrogen Bond
Unfavorable Acceptor-Acceptor
Alkyl
Pi-Alkyl
I2
Interactions
van der Waals
Conventional Hydrogen Bond
Pi-Anion
Pi-Sigma
Pi-Alkyl
J1
LEU E:61
GLN E:55
VAL E:101
TYR E:54
GLU E:50
ILE E:51
PHE E:104
Interactions
van der Waals
Carbon Hydrogen Bond
Alkyl
Pi-Alkyl
J2
Interactions
van der Waals
Conventional Hydrogen Bond
Unfavorable Donor-Donor
Pi-Donor Hydrogen Bond
Pi-Pi Stacked
Alkyl
Pi-Alkyl

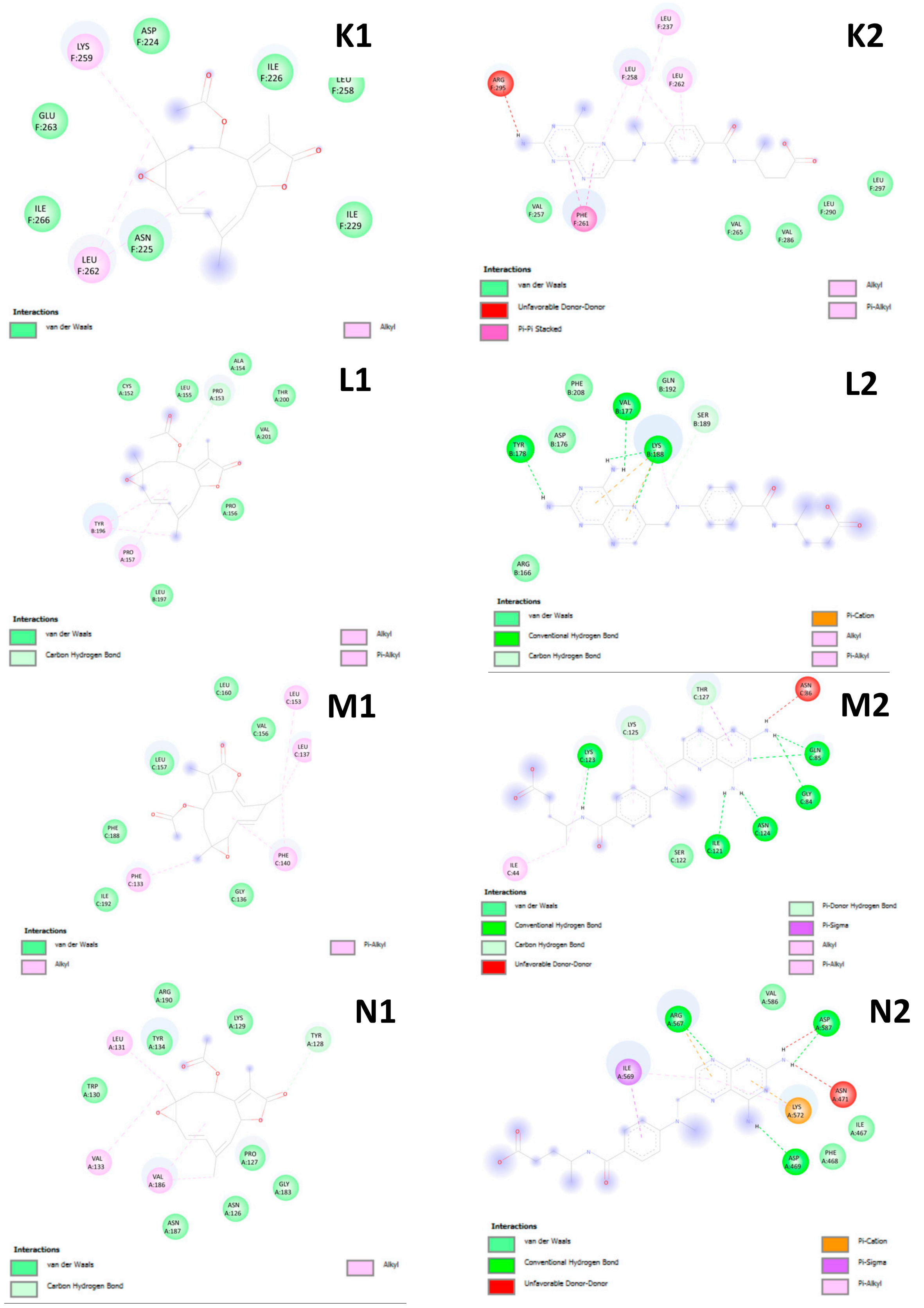

K1
LYS F:259
ASP F:224
ILE F:226
LEU F:258
GLU F:263
ILE F:266
ASN F:225
LEU F:262
ILE F:229
Interactions
van der Waals
Alkyl
K2
LEU F:237
LEU F:258
LEU F:262
ARG F:295
VAL F:257
PHE F:261
VAL F:265
VAL F:286
LEU F:290
LEU F:297
Interactions
van der Waals
Unfavorable Donor-Donor
Pi-Pi Stacked
Alkyl
Pi-Alkyl
L1
Interactions
van der Waals
Carbon Hydrogen Bond
Alkyl
Pi-Alkyl
L2
Interactions
van der Waals
Conventional Hydrogen Bond
Carbon Hydrogen Bond
Pi-Cation
Alkyl
Pi-Alkyl
M1
Interactions
van der Waals
Alkyl
Pi-Alkyl
M2
Interactions
van der Waals
Conventional Hydrogen Bond
Carbon Hydrogen Bond
Unfavorable Donor-Donor
Pi-Donor Hydrogen Bond
Pi-Sigma
Alkyl
Pi-Alkyl
N1
Interactions
van der Waals
Carbon Hydrogen Bond
Alkyl
N2
Interactions
van der Waals
Conventional Hydrogen Bond
Unfavorable Donor-Donor
Pi-Cation
Pi-Sigma
Pi-Alkyl

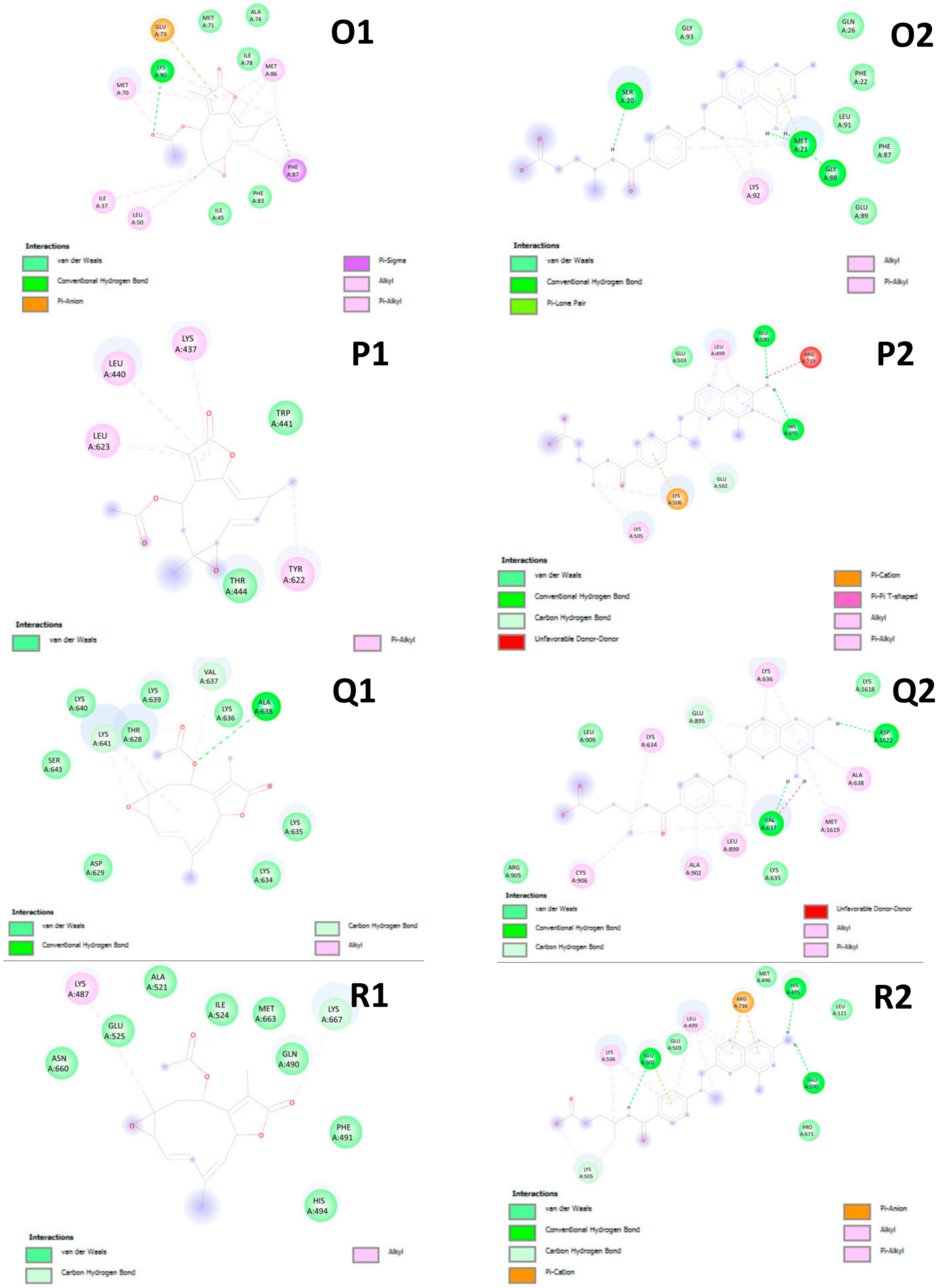
O1
GLU A:73
MET A:71
ALA A:74
LYS A:90
ILE A:78
MET A:86
MET A:70
PHE A:87
ILE A:37
LEU A:50
ILE A:45
PHE A:83
Interactions
van der Waals
Conventional Hydrogen Bond
Pi-Anion
Pi-Sigma
Alkyl
Pi-Alkyl
O2
GLY A:93
GLN A:26
SER A:20
PHE A:22
LEU A:91
MET A:21
PHE A:87
GLY A:88
LYS A:92
GLU A:89
Interactions
van der Waals
Conventional Hydrogen Bond
Pi-Lone Pair
Alkyl
Pi-Alkyl
P1
LYS A:437
LEU A:440
TRP A:441
LEU A:623
THR A:444
TYR A:622
Interactions
van der Waals
Pi-Alkyl
P2
GLU A:500
GLU A:503
LEU A:499
ARG A:719
HIS A:495
GLU A:502
LYS A:506
LYS A:505
Interactions
van der Waals
Conventional Hydrogen Bond
Carbon Hydrogen Bond
Unfavorable Donor-Donor
Pi-Cation
Pi-Pi T-shaped
Alkyl
Pi-Alkyl
Q1
VAL A:637
LYS A:640
LYS A:639
ALA A:638
LYS A:636
LYS A:641
THR A:628
SER A:643
LYS A:635
ASP A:629
LYS A:634
Interactions
van der Waals
Conventional Hydrogen Bond
Carbon Hydrogen Bond
Alkyl
Q2
LYS A:636
LYS A:1618
GLU A:895
LEU A:909
LYS A:634
ASP A:1622
ALA A:638
VAL A:637
MET A:1619
LEU A:899
ARG A:905
CYS A:906
ALA A:902
LYS A:635
Interactions
van der Waals
Conventional Hydrogen Bond
Carbon Hydrogen Bond
Unfavorable Donor-Donor
Alkyl
Pi-Alkyl
R1
LYS A:487
ALA A:521
ILE A:524
MET A:663
LYS A:667
GLU A:525
ASN A:660
GLN A:490
PHE A:491
HIS A:494
Interactions
van der Waals
Carbon Hydrogen Bond
Alkyl
R2
MET A:496
HIS A:495
ARG A:716
LEU A:121
LEU A:499
GLU A:503
LYS A:506
GLU A:502
GLU A:500
PRO A:671
LYS A:505
Interactions
van der Waals
Conventional Hydrogen Bond
Carbon Hydrogen Bond
Pi-Cation
Pi-Anion
Alkyl
Pi-Alkyl

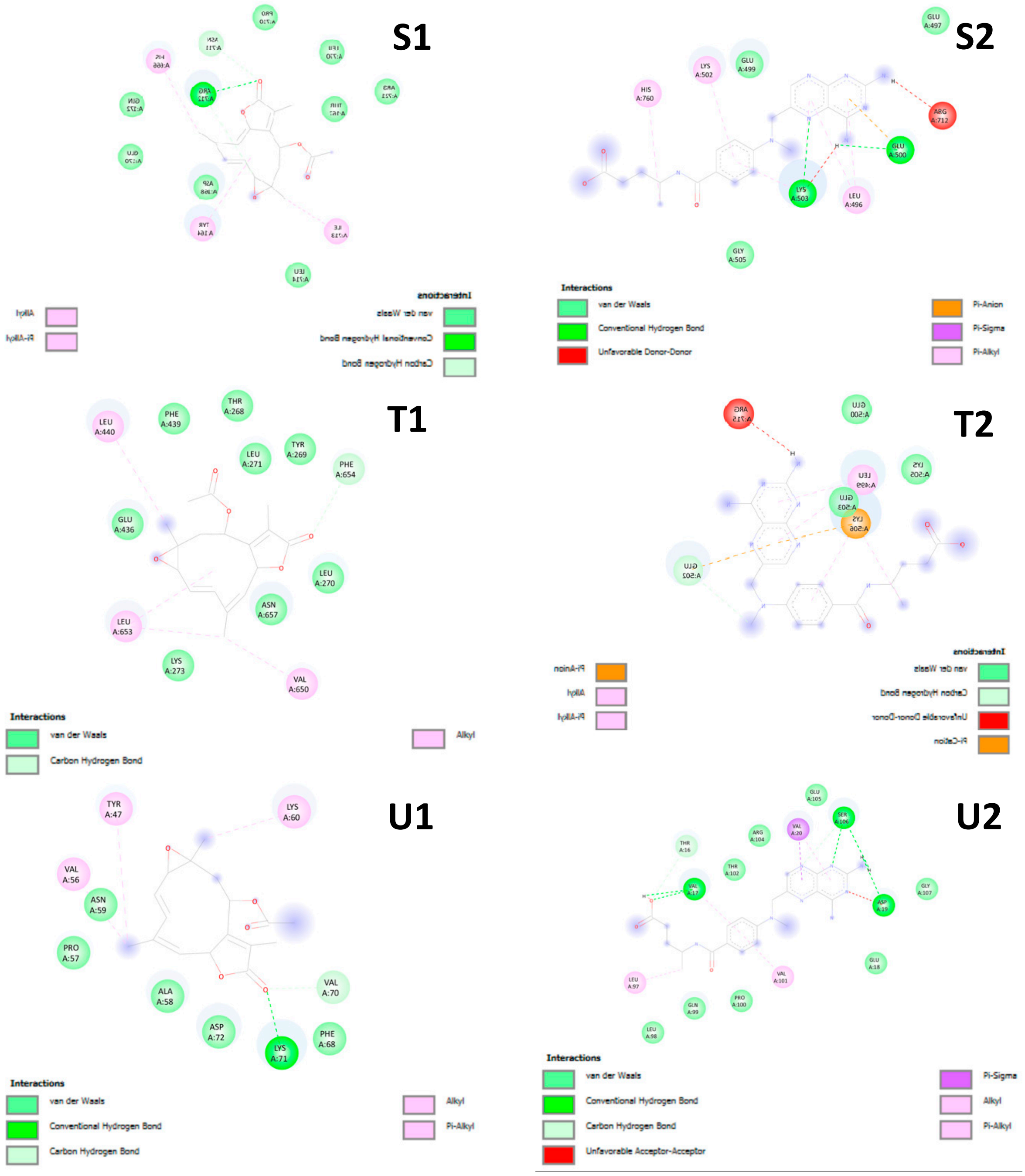
S1
S2
Interactions
van der Waals
Conventional Hydrogen Bond
Unfavorable Donor-Donor
Pi-Anion
Pi-Sigma
Pi-Alkyl
T1
T2
Interactions
van der Waals
Carbon Hydrogen Bond
Alkyl
U1
U2
Interactions
van der Waals
Conventional Hydrogen Bond
Carbon Hydrogen Bond
Alkyl
Pi-Alkyl
Interactions
van der Waals
Conventional Hydrogen Bond
Carbon Hydrogen Bond
Unfavorable Acceptor-Acceptor
Pi-Sigma
Alkyl
Pi-Alkyl

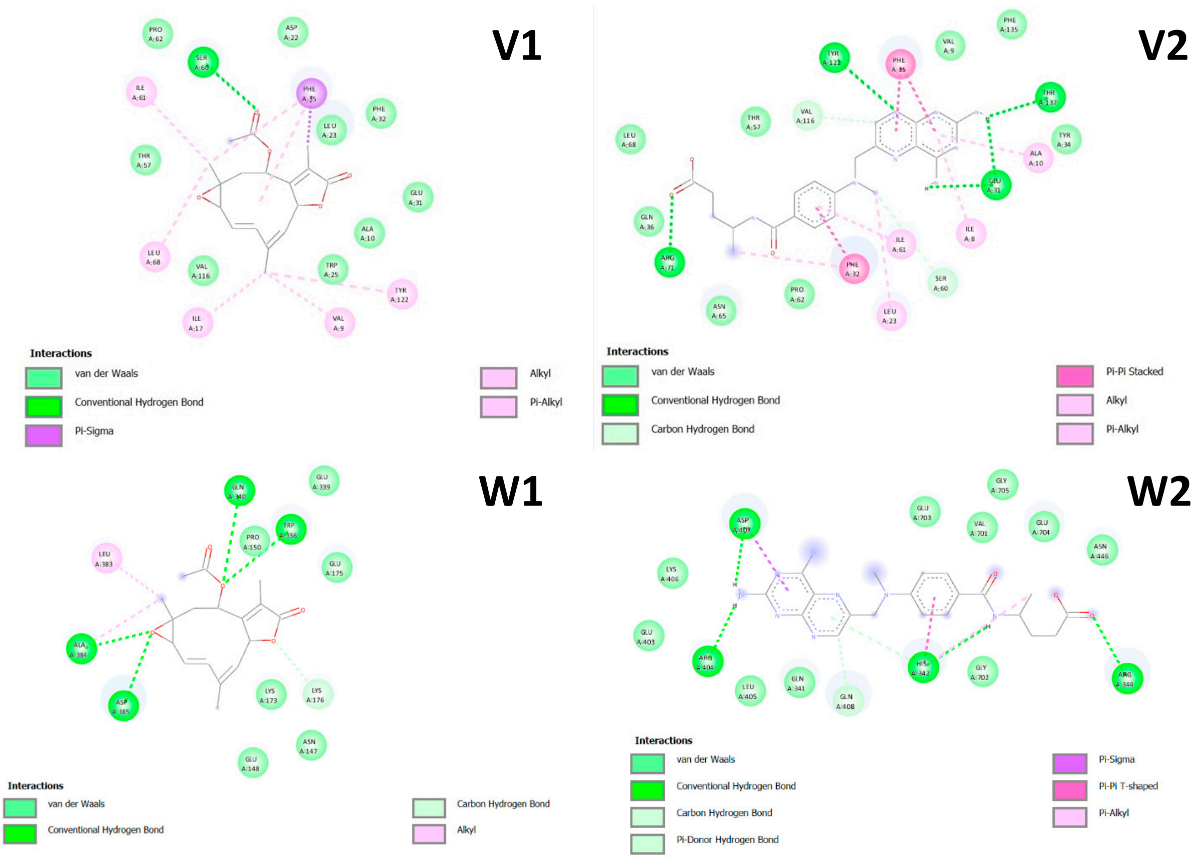

Figure S1. Results of molecular docking on proteins, 2D representation of interactions between Incomptine A (**1**) and Methotrexate (**2**) in **A)** ADP/ATP translocase, **B)** Alpha-actin3, **C)** Myozenin-1, **D)** 28S ribosomal protein S22, **E)** Actin, alpha skeletal muscle, **F)** Alpha-actin-2 , **G)** Beta-enolase, **H)** Gamma enolase (Eno2), **I)** Histone H2AX, **J)** Histone H3.3, **K)** Histone H4 (Hist1h4a or H4c11), **L)** Methyl-CPG-binding protein 2, **M)** Myosin light chain 3, **N)** Myosin regulatory light chain 2, **O)** Myosin regulatory light chain 2 Skeletal muscle, **P)** Myosin-1, **Q)** Myosin-3 (Myh-3), **R)** Myosin-4, **S)** Myosin-7, **T)** Myosin-8 , **U)** Myosin-binding protein C, fast-type, **V)** Dihydrofolate reductase, and **W)** Monofunctional C1-tetrahydrofolate dehydrogenase/cyclohydrolase.
